# Collateral Sensitivity Interactions between Antibiotics Depend on Local Abiotic Conditions

**DOI:** 10.1128/mSystems.01055-21

**Published:** 2021-11-30

**Authors:** Richard C. Allen, Katia R. Pfrunder-Cardozo, Alex R. Hall

**Affiliations:** a Department of Environmental Systems Science, ETH Zürich, Zürich, Switzerland; University of Illinois at Chicago

**Keywords:** antibiotic resistance, collateral sensitivity

## Abstract

Mutations conferring resistance to one antibiotic can increase (cross-resistance) or decrease (collateral sensitivity) resistance to others. Antibiotic combinations displaying collateral sensitivity could be used in treatments that slow resistance evolution. However, lab-to-clinic translation requires understanding whether collateral effects are robust across different environmental conditions. Here, we isolated and characterized resistant mutants of Escherichia coli using five antibiotics, before measuring collateral effects on resistance to other paired antibiotics. During both isolation and phenotyping, we varied conditions in ways relevant in nature (pH, temperature, and bile). This revealed that local abiotic conditions modified expression of resistance against both the antibiotic used during isolation and other antibiotics. Consequently, local conditions influenced collateral sensitivity in two ways: by favoring different sets of mutants (with different collateral sensitivities) and by modifying expression of collateral effects for individual mutants. These results place collateral sensitivity in the context of environmental variation, with important implications for translation to real-world applications.

**IMPORTANCE** When bacteria become resistant to an antibiotic, the genetic changes involved sometimes increase (cross-resistance) or decrease (collateral sensitivity) their resistance to other antibiotics. Antibiotic combinations showing repeatable collateral sensitivity could be used in treatment to slow resistance evolution. However, collateral sensitivity interactions may depend on the local environmental conditions that bacteria experience, potentially reducing repeatability and clinical application. Here, we show that variation in local conditions (pH, temperature, and bile salts) can influence collateral sensitivity in two ways: by favoring different sets of mutants during bacterial resistance evolution (with different collateral sensitivities to other antibiotics) and by modifying expression of collateral effects for individual mutants. This suggests that translation from the lab to the clinic of new approaches exploiting collateral sensitivity will be influenced by local abiotic conditions.

## INTRODUCTION

As a result of antibiotic use, resistance is increasing in bacteria (1), necessitating efforts to identify new antibiotic types (2). However, the time, money, and risks involved in getting new therapeutics to the clinic (3) and the targeting of many essential bacterial pathways by existing antibiotics mean that the rate of development for new antibiotics is outstripped by rates of resistance development (3). To tackle the threat of antibiotic resistance, we must investigate strategies to slow the spread of resistance to existing treatments and to any new treatments in development (4). One strategy that shows promise in slowing the evolution of resistance is to exploit collateral sensitivity interactions (5–8). These have been observed for specific combinations of antibiotics where mutations conferring resistance to one antibiotic sensitize bacteria to a second antibiotic (5–8), thereby increasing the effectiveness of the second antibiotic and reducing the potential for resistance evolution to it (6, 9). For collateral sensitivity interactions to be exploited therapeutically, it is important that their emergence across different populations of bacteria, such as those in different patients or in different communities, be repeatable. That is, unless collateral sensitivity interactions are predictable, exploiting them in new treatment strategies will be very challenging (10–13).

Recent work revealed important genetic factors influencing the predictability of collateral sensitivity, but the importance of local abiotic conditions is still unclear. For example, high-throughput *in vitro* studies showed that different replicate populations exposed to the same antibiotic sometimes acquire collateral sensitivity to another antibiotic and sometimes do not (10, 11). This can be explained by different mutations, which vary in their phenotypic effects on resistance, spreading in different replicate populations (11, 12, 14, 15). However, we know from past work that phenotypic effects of antibiotic resistance mechanisms also vary strongly depending on local environmental conditions (16–19). For example, bile can upregulate efflux pumps (20), zinc can reduce the activity of aminoglycoside-degrading enzymes (21), and high temperature can modulate the effects of rifampicin resistance mutations on growth in the absence of antibiotics (22). This raises the possibility that local environmental conditions could influence the emergence of collateral sensitivity by affecting which of the possible pathways to resistance are most strongly selected, both in the absence of antibiotics and during antibiotic exposure. Furthermore, the abiotic environment could affect the expression of collateral effects, by modifying the phenotypic effects of resistance alleles when bacteria are exposed to a second antibiotic. To date, research on collateral sensitivity interactions has focused on testing many combinations of antibiotics (5, 6, 9, 14), multiple strains (10), or many replicate populations for individual antibiotic combinations (11). Therefore, the role of local abiotic conditions in the emergence and expression of collateral sensitivity interactions remains unclear. Answering this question would improve our understanding of the robustness of collateral sensitivity across different populations and environments. This would in turn boost our ability to predict pathogen responses to treatment regimens that exploit collateral sensitivity interactions.

To address these gaps in our knowledge, we tested for collateral effects (cross-resistance or collateral sensitivity) between five pairs of antibiotics, each in four different experimental environments. Each antibiotic pair consisted of a selection antibiotic (which we used in mutant isolation) and a paired antibiotic (which we used to test for collateral effects). We chose pairs of antibiotics indicated by past work to at least sometimes display collateral sensitivity interactions (5, 6). The four experimental environments were (i) basal, nutrient-rich broth (lysogeny broth [LB] at 37°C and buffered at pH 7.0), plus three types of abiotic environmental variation which we expect to be relevant to pathogens *in vivo*; (ii) reduced pH (pH 6.5), as found in certain body compartments, including abscesses and parts of the gastrointestinal (GI) tract (23, 24); (iii) increased temperature (42°C), as found in companion and livestock animals with higher core temperatures than humans (25); and (iv) the presence of bile salts (0.5 g/L bile salts), which bacteria must contend with in the GI tract (26).

In each of four sets of abiotic conditions, we grew multiple independent populations of Escherichia coli K-12 MG1655 in the absence of antibiotics, before screening for antibiotic-resistant mutants, on agar with the selection antibiotic (shown schematically in Fig. S1 in the supplemental material). From these resistant mutants, we randomly chose single colonies (from independent populations) to isolate and sequence. For each isolate, we then measured resistance as the 90% inhibitory concentration (IC_90_) for the relevant selection antibiotic and paired antibiotic, again across all four sets of abiotic conditions (Fig. S1). Unlike past work, this manipulation of the experimental environment, during both isolation and phenotyping, in a fully factorial design allowed us to quantify the effects of local abiotic conditions on the emergence (which mutations are selected in which treatments?) and expression (in which abiotic environments do we see collateral effects from particular mutants?) of collateral sensitivity for multiple candidate antibiotic pairs. Our aim was to quantify the effect of environmental conditions on collateral sensitivity interactions for a range of different mutants, rather than to focus on individual mutations. Nevertheless, for some of the mutations we identified, existing information about their physiological effects provides mechanistic insight into the origins of observed collateral effects, and we place our findings in this context where relevant.

10.1128/mSystems.01055-21.1 FIG S1 Schematic of workflow, showing a single mutant selected for cefuroxime resistance. (a) The ancestral strain (E. coli K-12 MG1655) was grown in liquid cultures (without antibiotics) overnight. (b) The cultures were transferred to agar with the same environmental conditions as during overnight growth (only basal conditions are shown here) and the selection antibiotic at selection concentration. If there was growth after 48 h (resistant mutants), single colonies were picked and subjected to whole-genome sequencing (c). (d) For each mutant, dose response curves were measured against the selection antibiotic and a paired antibiotic in all four abiotic environments (assay environment; mutants from all selection environments measured in all assay environments). From these curves we could calculate the antibiotic-free growth, the IC_90_ of the selection antibiotic, the IC_90_ of the paired antibiotic, and the growth at selection concentration (selection antibiotic only) for each mutant in each assay environment. (e) The same workflow was used for the other four antibiotic pairs, but using the selection and paired antibiotics given in the table. Although we show only single replicates here, we grew and selected 92 independent populations for each environment-antibiotic combination (1,840 total). We randomly chose up to 6 mutants to sequence and phenotype for each combination (Table S2). Download FIG S1, TIF file, 0.9 MB. Copyright © 2021 Allen et al. 2021 Allen et al. https://creativecommons.org/licenses/by/4.0/ This content is distributed under the terms of the Creative Commons Attribution 4.0 International license.

## RESULTS

### Different resistance mutations isolated depending on local abiotic conditions.

**FIG 1:**
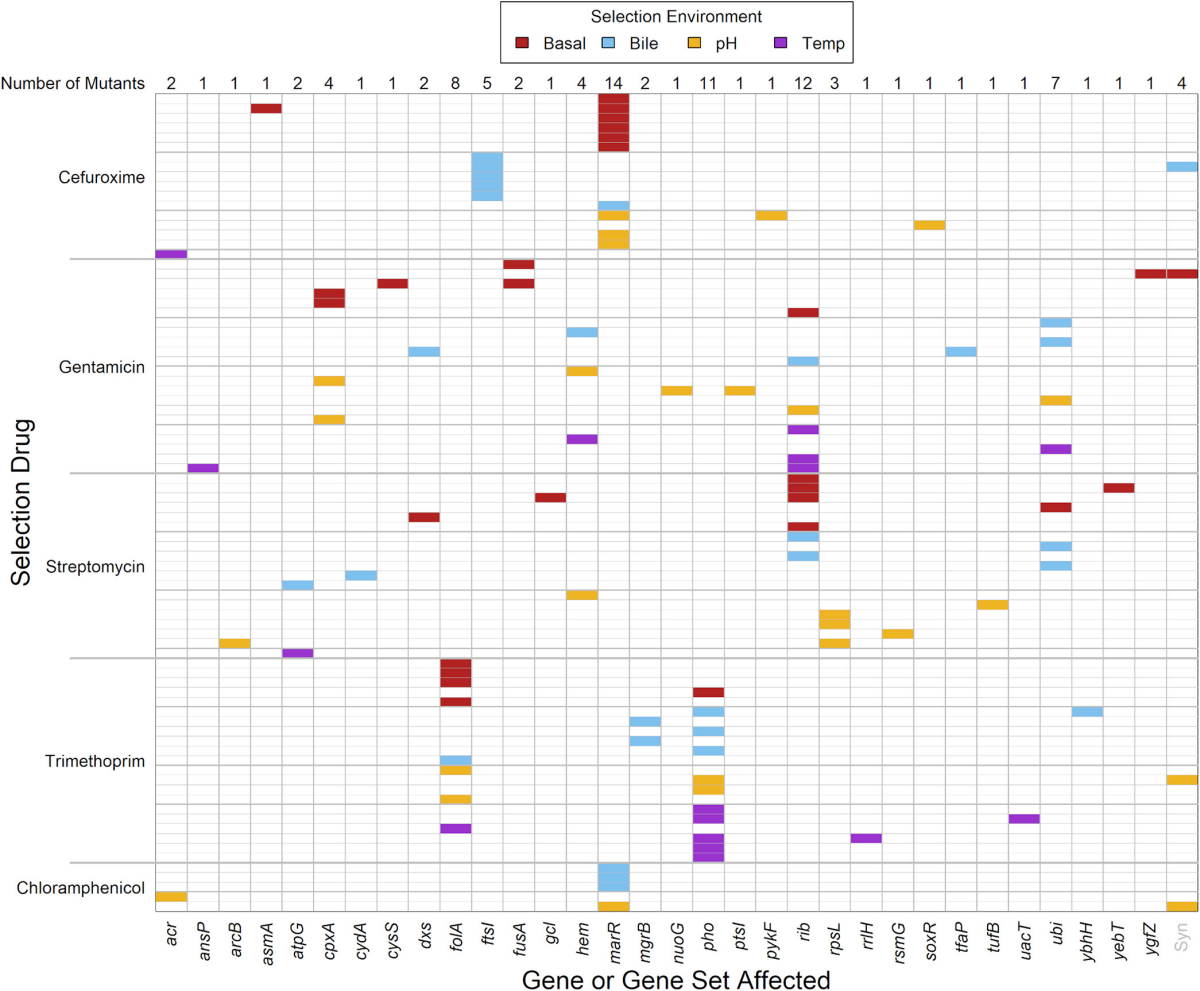
Genes affected by mutations, in mutants selected for resistance to different antibiotics in different selection environments. Each row of cells represents one mutant; rows are grouped by which antibiotic they were selected against (selection drug; labeled at left) and colored by the abiotic conditions during selection (selection environment; legend at top with the following abbreviations: basal, basal conditions of pH 7.0 and 37°C; bile, with added bile salts at 0.5 g/L; pH, with reduced pH of 6.5; temp, increased temperature of 42°C). Each column of cells represents one gene or gene set that was affected by mutations in our isolated mutants (ordered alphabetically). Each gene set comprises multiple genes involved in the same pathway (see Materials and Methods); these were *acr*, *hem*, *pho*, *rib*, and *ubi*. At the top, the total number of mutants with mutations affecting each gene is shown. Sixteen of the 84 mutants contained multiple mutations affecting multiple genes, all of which are shown. The rightmost column (Syn) shows synonymous mutations, found in four mutants (all four mutants also had other, nonsynonymous mutations). For more information, see Table S1.

We used whole-genome sequencing to identify genetic changes relative to the ancestral strain for 84 resistant mutants (Fig. 1; Table S1), each isolated after exposure to one of five antibiotics (selection antibiotic, defined as the antibiotic present in the agar plate used to select resistant mutants from ancestral cells) in four different abiotic environments (selection environment, defined as the local abiotic conditions during overnight growth prior to plating and in the agar plate). We found that mutants selected with the same selection antibiotic tended to have mutations affecting the same genes more often than mutants selected with different selection antibiotics (permutational multivariate analysis of variance [PERMANOVA]: *F*_3,72_ = 7.21; *P* < 0.001). Similarly, mutants selected in the same selection environment tended to have mutations affecting the same genes more often than mutants selected in different selection environments (PERMANOVA: *F*_3,75_ = 2.44; *P* < 0.001). Looking at each selection antibiotic separately, there was an effect of selection environment for cefuroxime (*F*_3,13_ = 7.50; corrected *P* < 0.01) and streptomycin (*F*_3,15_ = 2.19; corrected *P* < 0.05) but not trimethoprim and gentamicin (corrected *P* > 0.05). For streptomycin, the major differences were between the mutations selected in the low-pH environment and those in other selection environments. For cefuroxime, we found mutations in the penicillin-binding protein gene *ftsI*, but only in the presence of bile (Fig. 1). For chloramphenicol, we obtained genotypic information for only five mutants; these are shown in Fig. 1 and Fig. S2 but not included in the analyses here or below because of the much smaller sample size. In summary, the types of resistance mechanisms that were selected during our mutant screen depended on the local abiotic conditions.

**FIG 2:**
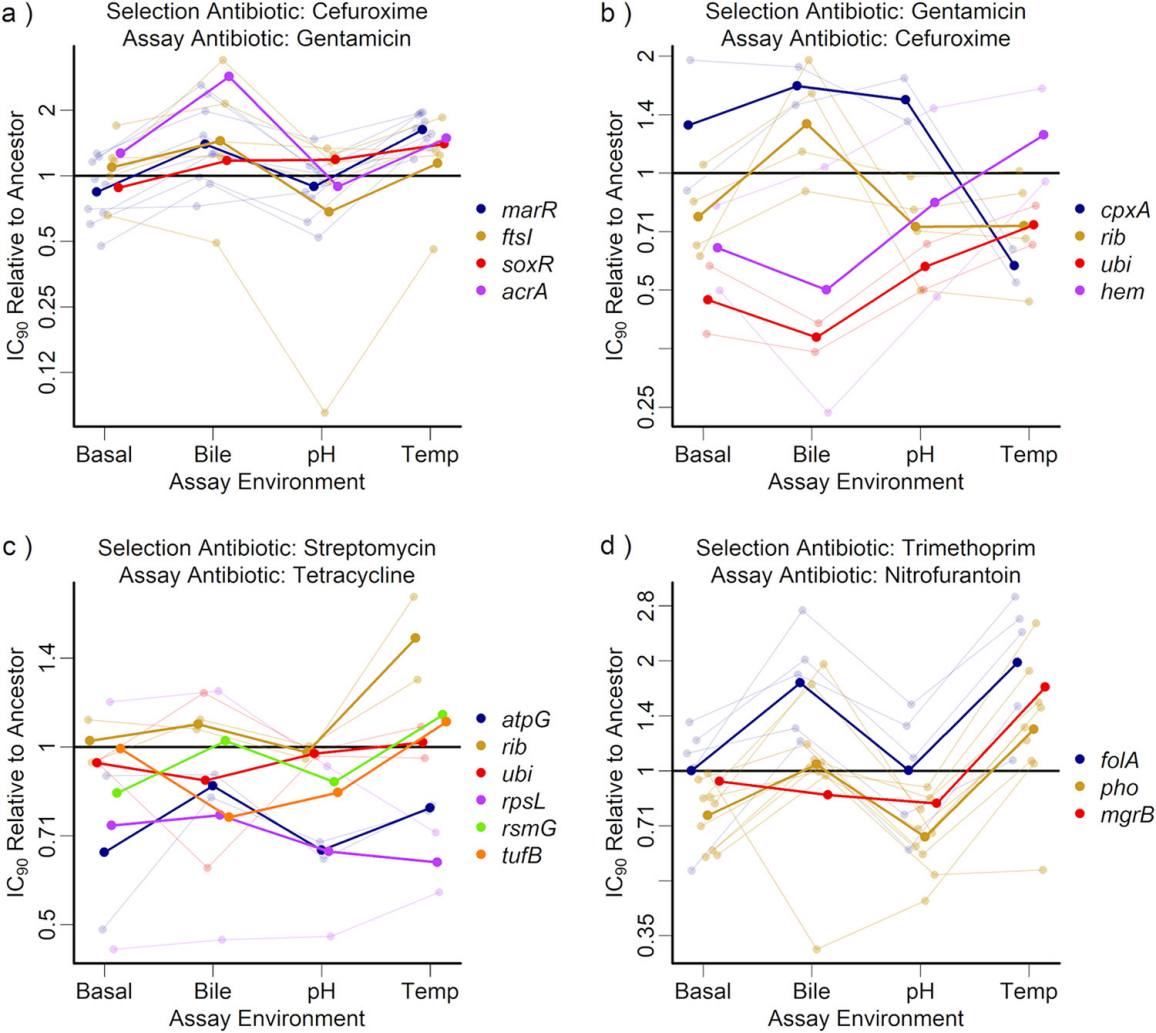
Collateral changes in resistance to a paired antibiotic for resistant mutants selected with each selection antibiotic, tested across different abiotic conditions (*x* axis, assay environments). Each panel shows mutants selected for resistance to a selection antibiotic and then tested for resistance to a paired antibiotic (labeled at the top of the panel). The IC_90_ for each mutant of the paired drug is shown relative to the ancestor in the same environment (scores for the ancestor are given in Fig. S3): mutants with points of >1 are more resistant and those with points of <1 are more sensitive than the ancestor. Mutants are grouped and colored according to the gene or gene set that was mutated; the thick lines show mean IC_90_ across all mutants with the same gene/gene set mutated. Where there were multiple mutants with the same gene/gene set mutated, thin lines show IC_90_s for the individual mutants. The *y* axis is log transformed, and the scale varies between panels.

**FIG 3:**
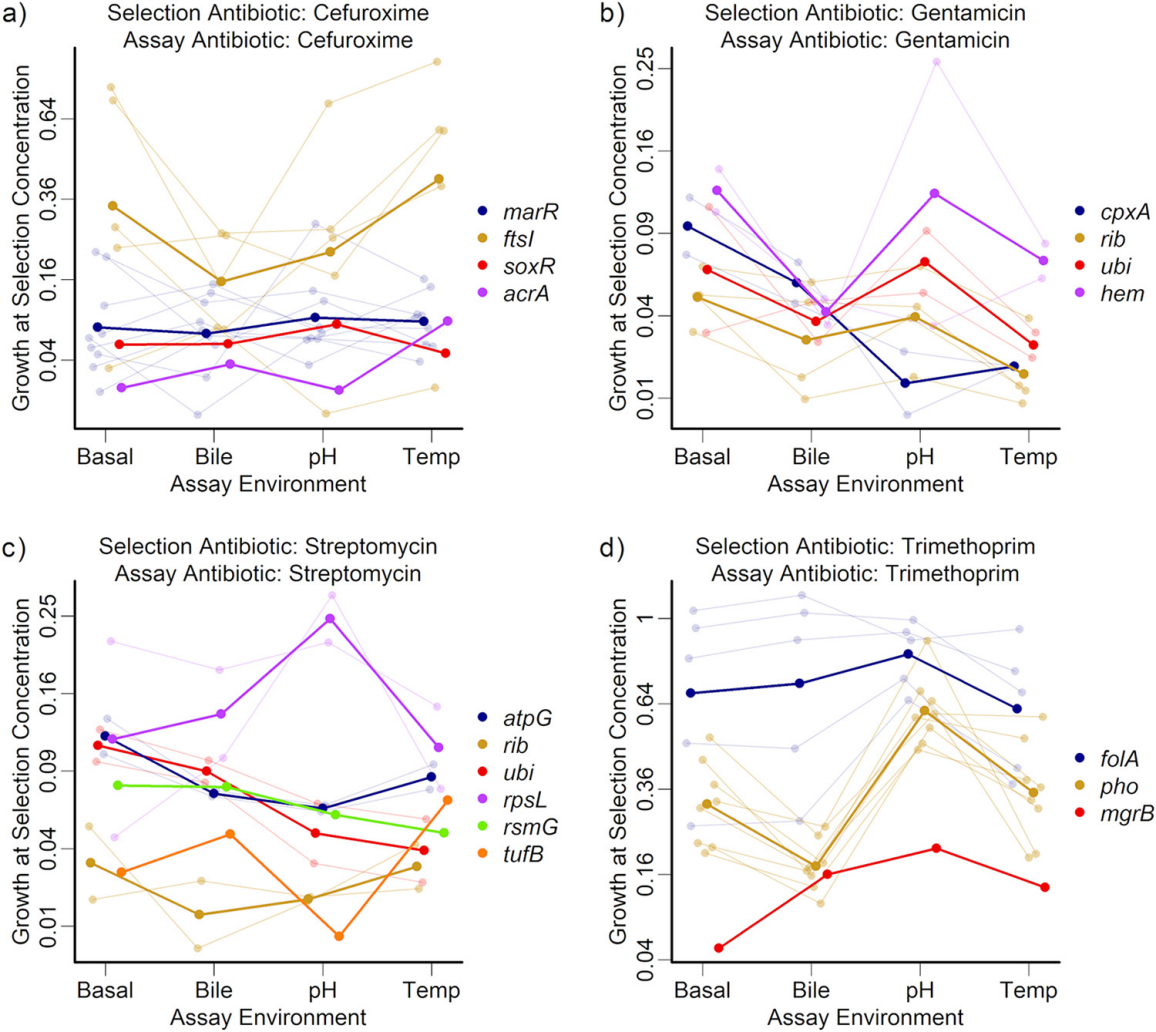
Population growth for resistant mutants selected with each selection drug when grown with that antibiotic at selection concentration (GASC), measured in four different assay environments. Each panel shows mutants selected for resistance to the selection antibiotic shown in the panel title and then assayed for growth at the selection concentration of that drug. Mutants are grouped and colored according to the gene or gene set that was mutated, with the thick lines showing the mean GASC across all strains with the same genotype. Where there were multiple mutants with the same genotype, thin lines show the GASC for the individual mutants. The *y* axis is square-root transformed and varies between panels.

**FIG 4:**
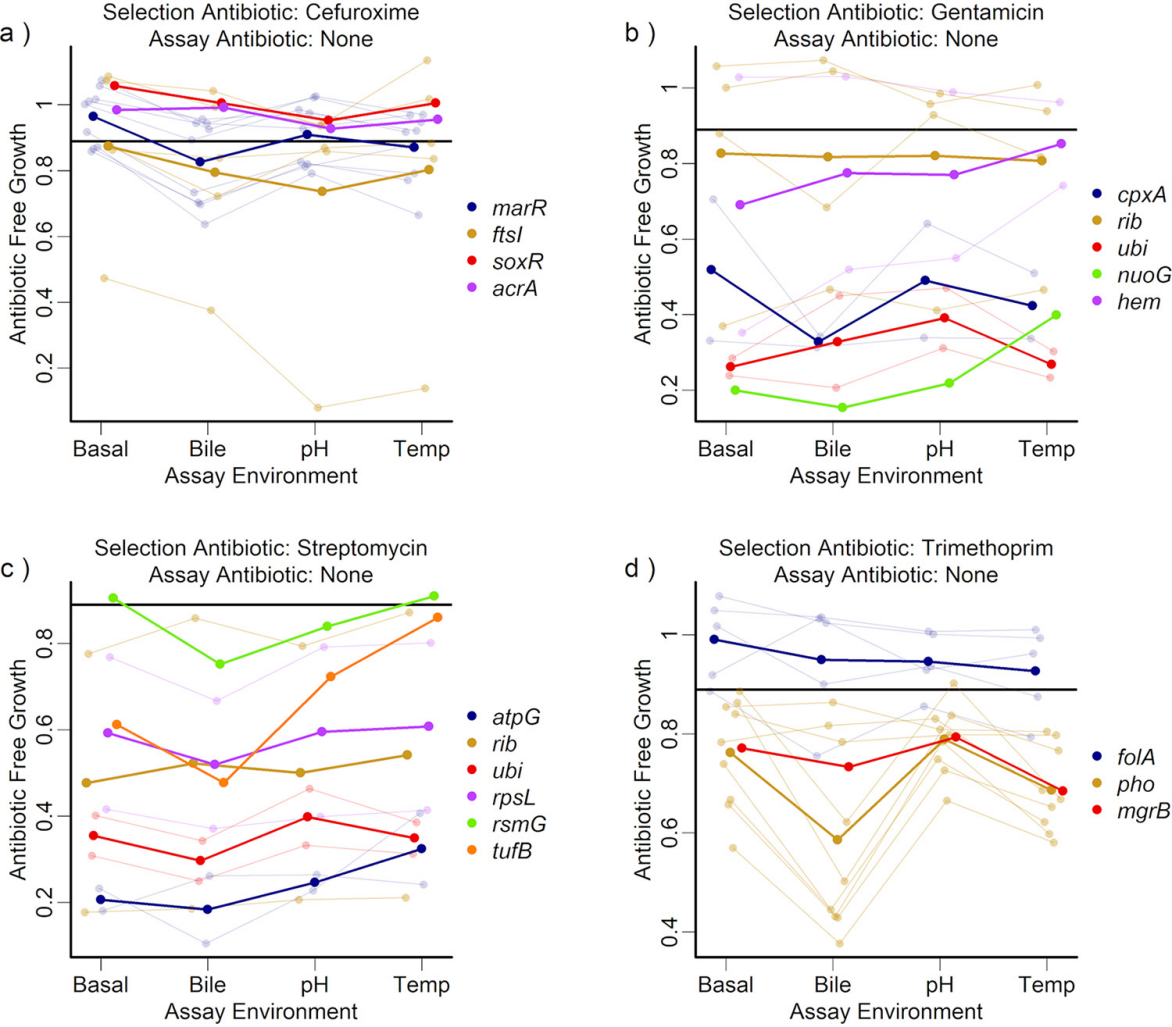
Antibiotic-free growth of resistant mutants selected with each selection antibiotic, measured in four different assay environments. Each panel shows the antibiotic-free growth for mutants selected for resistance to one of four selection drugs, as indicated for each panel. Mutants are grouped and colored according to the gene or gene set that was mutated, with the thick lines showing the mean antibiotic-free growth across all strains with the same genotype. Where there were multiple mutants with the same genotype, thin lines show the antibiotic-free growth for the individual mutants. The black horizontal line in each plot gives the mean antibiotic-free growth of the ancestral strain (across all assay environments). The *y* axis scale varies between panels.

10.1128/mSystems.01055-21.2 FIG S2 Phenotypes for chloramphenicol selected strains. (a) Nitrofurantoin resistance; (b) chloramphenicol resistance; (c) GASC of chloramphenicol; (d) antibiotic-free growth for the isolates resistant to chloramphenicol, where we have genotyping information. Thick lines show the mean phenotypic value for the 4 strains with mutations in *marR* and the 1 strain with mutations in *acrR*. The phenotypic values of the 4 *marR* mutants are shown as thin blue lines. The phenotypic value for the ancestor is shown as a horizontal black line. The *y* axes in panels a and b are log transformed, and the *y* axis in panel c is square root transformed (as for Fig. 2 to 4). Mutated genes influenced IC_90_ of chloramphenicol (*F*_2,38_ = 25.8; *P* < 0.001), GASC (*F*_2,78_ = 48.6; *P* < 0.001), and antibiotic-free growth (*F*_2,30_ = 632; *P* < 0.001). Mutated genes interacted with environment to influence IC_90_ of chloramphenicol (χ^2^_3_ = 20.4; *P* < 0.001), GASC (*F*_3,72_ = 3.08; *P* < 0.05), and antibiotic-free growth (χ^2^_3_ = 8.68; *P* < 0.05). Download FIG S2, TIF file, 0.3 MB. Copyright © 2021 Allen et al. 2021 Allen et al. https://creativecommons.org/licenses/by/4.0/ This content is distributed under the terms of the Creative Commons Attribution 4.0 International license.

10.1128/mSystems.01055-21.8 TABLE S1 Mutations identified in the 110 strains genotyped. Mutations were identified by breseq, run in consensus mode, using the genotype of the ancestral strain as a reference. All mutations detected by breseq are shown. “Sample Code” is shorthand for each mutant (e.g., CFR1Basal is the first cefuroxime-resistant mutant isolated with the selection drug cefuroxime and the selection environment basal). Antibiotic abbreviations: CFR, cefuroxime; CHL, chloramphenicol; GEN, gentamicin; STR, streptomycin; TRM, trimethoprim. “Position” is the position in the genome that was mutated (relative to the E. coli K-12 MG1655 reference) or the start position for multi-base-pair mutations. “Gene” is the gene(s) that was mutated; where there are two genes separated by a slash, the mutation occurred between them. The number in brackets represents either the nucleotide position from the start of the gene for mutations in coding regions or the distances to the neighboring genes in the case of intergenic mutations (minus and plus signs indicate upstream and downstream). “Gene mutated” is the gene influenced by the mutation, either because the mutation occurred in that gene or because the mutation affects the promoter region of that gene (as determined by breseq). Genes with a superscript 1 were intergenic mutations where breseq did not assign a gene, but we manually identified which promoter was influenced based on promoter information (https://ecocyc.org/). Genes with a superscript 2 are synonymous mutations; these are listed here and in Fig. 1 but were not used for any analysis. Sixteen strains had two or three mutations influencing two different genes and thus have two or three consecutive rows in the table. For the phenotypic analysis, each isolate could be assigned to only one group. Therefore, genes with a superscript 3 are genes that were not used to group the mutations in the phenotyping analysis (see Materials and Methods). These genes were still used in the PERMANOVA, where multiple mutations per isolate could be included. “Gene Set Affected” is the gene set used for both the PERMANOVA genome models and the phenotypic analysis, blank entries are synonymous mutations (superscript 2 above) not used for either analysis, and entries in brackets were used only in the PERMANOVA (superscript 3 above). “Details” are given regarding the type of SNP and amino acid change, if relevant, or the size of deletion and mobilization events. “Coverage” gives the average coverage across the entire genome of the isolate; where there are multiple mutations, the same value is given for each mutation. STR6pH contained a large deletion affecting multiple genes; of these, only *hemB* was mutated in other isolates, so we assigned this large deletion as affecting *hemB*. In some isolates, we could not identify any mutations, so no mutations information is given, but coverage is still reported. We were able to identify mutations in 84 of the strains. Download Table S1, DOCX file, 0.1 MB. Copyright © 2021 Allen et al. 2021 Allen et al. https://creativecommons.org/licenses/by/4.0/ This content is distributed under the terms of the Creative Commons Attribution 4.0 International license.

### Collateral sensitivity and cross-resistance vary depending on resistance mechanism.

We tested whether different resistant mutants showed variable susceptibility to paired antibiotics previously implicated in collateral sensitivity. For the selection antibiotics cefuroxime, gentamicin, streptomycin, and trimethoprim, the paired antibiotics were gentamicin (6), cefuroxime (6), tetracycline (5), and nitrofurantoin (5), respectively. The average fold change in IC_90_ of paired antibiotics was low (mean ± standard deviation of log_2_-transformed relative IC_90_, 0.01 ± 0.71) (Fig. 2 and Fig. S3) compared to changes in resistance to the selection antibiotics (1.99 ± 1.13) (Fig. 2 and Fig. S3; discussed further below) and encompassed both positive (cross-resistance) and negative (collateral sensitivity) changes in resistance (Fig. 2). For individual pairs of antibiotics, average collateral effects varied depending on which gene was mutated for the sets of mutants selected against gentamicin and then tested against cefuroxime (main effect of genotype on cefuroxime IC_90_: *F*_4,46_ = 6.73; corrected *P* < 0.01), mutants selected against streptomycin and tested against tetracycline (*F*_5,139_ = 3.93; corrected *P* < 0.01), and mutants selected against trimethoprim and tested against nitrofurantoin (χ^2^_2_ = 10.8; corrected *P* < 0.01) but not for mutants selected against cefuroxime and tested against gentamicin (*F*_4,19_ = 0.189, corrected *P* > 0.5). We found several genes that were consistently associated with collateral sensitivity to paired antibiotics (shown by negative values of log_2_-transformed relative IC_90_), such as *ubi* mutants, which were on average collaterally sensitive to cefuroxime (effect of *ubi* mutation on log_2_-transformed relative IC_90_: β = −0.98: *t*_38_ = 3.51; *P* < 0.001) (Fig. 2b), and *atpG* mutants, which were on average collaterally sensitive to tetracycline (β = −0.452; *t*_148_ = 3.27; *P* < 0.01) (Fig. 2c). Mutations in *ubi* genes and in *atpG* affect ubiquinone synthesis and the ATP synthase, respectively, disrupting the proton motive force (PMF), which in turn leads to a reduced membrane potential and hence reduced influx of aminoglycosides (27). Despite the benefit of aminoglycoside resistance, PMF-driven efflux pumps such as acrAB are less active in mutants with disrupted PMF (5), increasing susceptibility to other antibiotics.

10.1128/mSystems.01055-21.3 FIG S3 IC_90_ of antibiotics compared across directly and indirectly selected mutants and ancestral strain. Panels show resistance (IC_90_) to (a) cefuroxime, (b) gentamicin, (c) streptomycin, (d) trimethoprim, (e) tetracycline, (f) nitrofurantoin, (g) chloramphenicol, and (h) polymyxin B in the 4 different abiotic environments for all relevant strains. Strains shown are mutants directly selected with the same antibiotic (red box plots), mutants selected with another antibiotic (i.e., collateral effects; blue box plots), and the ancestral strain (yellow lines). See keys in individual panels for the specific drugs used for selection of mutants. Box plots show medians and interquartile ranges across all individual mutants measured under the relevant conditions (where the score for each mutant is first averaged across replicate assays). Data for the ancestral strain are the means of all independent estimates of IC_90_ under the relevant conditions (mean *n *= 4.8); error bars show standard errors of the means. *y* axes are log transformed and vary between panels. For the ancestral strain, assay environment had the strongest effects on resistance to gentamicin (*F*_3,24_ = 12.5; uncorrected *P* < 0.001), streptomycin (*F*_3,8_ = 4.83; uncorrected *P* < 0.05), and polymyxin (χ^2^_3_ = 8.93; uncorrected *P* < 0.05), although only for gentamicin was this effect significant after accounting for multiple testing. All IC_90_ comparisons reported in the text are relative to the ancestral IC_90_ in the same abiotic environment. This could in theory influence the main effect of assay environment on collateral effects, as reported in the text (seen for the selection-paired drug combinations cefuroxime-gentamicin and trimethoprim-nitrofurantoin). However, we see the same main effect of assay environment in these two combinations when we use absolute IC_90_ of the paired drug for resistant mutants instead of IC_90_ relative to the ancestor (cefuroxime-gentamicin χ^2^_3_ = 126, corrected *P* < 0.001; trimethoprim-nitrofurantoin χ^2^_3_ = 9.51, corrected *P* < 0.05). We also see a significant effect for streptomycin-selected mutants (streptomycin-tetracycline χ^2^_3_ = 15.1, corrected *P* < 0.01), which was not significant using IC_90_ relative to the ancestor (see the text). Download FIG S3, TIF file, 0.5 MB. Copyright © 2021 Allen et al. 2021 Allen et al. https://creativecommons.org/licenses/by/4.0/ This content is distributed under the terms of the Creative Commons Attribution 4.0 International license.

For mutants selected against trimethoprim, the variable collateral effects observed for different mutants/genotypes translated to differences in average resistance to nitrofurantoin depending on which environment they were selected in (effect of selection environment on IC_90_ to nitrofurantoin: χ^2^_3_ = 20.6; corrected *P* < 0.01). Mutants selected at high temperature (β = −0.26; *t*_58_ = 4.19; *P* < 0.05) had significant collateral sensitivity. Mutations in *phoPQ*, which were associated with relatively high sensitivity to nitrofurantoin (Fig. 2d), were more common at high temperature than in the other selection environments (Fig. 1). In summary, mutants selected for resistance to one antibiotic often had altered average resistance to other antibiotics (as expected, as these antibiotics were chosen based on past evidence of such effects). However, these collateral effects varied among different pathways to resistance (mutated genes), which translated in some cases to variation of average collateral effects depending on the abiotic conditions during selection for resistance to the first antibiotic (selection environment).

### Collateral effects depend on the assay environment.

Expression of average collateral effects to paired antibiotics also varied depending on the abiotic conditions during exposure to these antibiotics. For mutants selected against cefuroxime and trimethoprim, there was a significant effect of assay environment on average resistance to the paired antibiotics, relative to the ancestor under the same conditions (effect of assay environment on resistance to paired antibiotic for selection-paired antibiotic: cefuroxime-gentamicin, χ^2^_3_ = 53.0, corrected *P* < 0.001; trimethoprim-nitrofurantoin, χ^2^_3_ = 89.1, corrected *P* < 0.001) (Fig. 2). Note that this variation of susceptibility relative to the ancestral strain was not explained by variable susceptibility of the ancestral strain across assay environments (Fig. S3). Qualitatively similar results emerge (see the legend to Fig. S3) if we use the absolute IC_90_ (not relative to the ancestor).

Changing the local abiotic conditions did not affect all mutants the same way: the mutated gene and assay environment interacted to determine resistance to cefuroxime in mutants selected against gentamicin (genotype-by-assay environment interaction effect on cefuroxime resistance: χ^2^_9_ = 38.3; corrected *P* < 0.001). For some mutants, this variation led to a switch between cross-resistance and collateral sensitivity, such as *cpxA* mutants (Fig. 2b), which were resistant to cefuroxime in the bile (β = 0.74; *t*_130_ = 2.48; *P* < 0.05) and pH (β = 0.63; *t*_130_ = 2.09; *P* < 0.05) environments but were susceptible to cefuroxime at high temperature (β = −0.79; *t*_130_ = 2.64; *P* < 0.01). *cpxA* is part of a two-component regulator which responds to misfolded proteins in the periplasm, activating the Cpx response, which has been shown to confer resistance to aminoglycosides (28). Mutations in the periplasmic domain of *cpxA* (as in our mutants) have the Cpx pathway locked into an activated state (29), with Cpx phenotypes being more pronounced at high temperature (30, 31). Other work has shown that, due to its influence on cell wall homeostasis, the Cpx response can influence resistance to β-lactams like cefuroxime but that it must be at an intermediate level for maximal resistance (32). At temperatures of 37°C, our mutants likely upregulate the Cpx response into the optimum zone, leading to β-lactam resistance. However, at 42°C, the mutant’s Cpx response is likely further upregulated (30, 31), meaning that peptidoglycan homeostasis is no longer maintained, resulting in β-lactam sensitivity (32). This shows that collateral effects for individual resistant strains can change qualitatively across different abiotic environments.

### Environment-dependent selection for particular genotypes is revealed by analyzing growth at the selecting antibiotic concentration but not resistance as IC_90_.

Having found that local abiotic conditions influenced collateral sensitivity by changing both the expression of resistance phenotypes for individual mutants and which mutants we isolated upon antibiotic exposure, we sought to explain why different mutants were isolated in different selection environments. The first possible explanation we tested was that the identity of the mutations conferring most effective resistance against each selection antibiotic (and therefore most likely to form a colony and be detected in our mutant screen) may depend on local abiotic conditions (Fig. S4). We found no statistical support for such an effect when we measured resistance (IC_90_) of each mutant to its corresponding selection antibiotic in all four abiotic conditions: assay environment did not significantly alter the observed variation of IC_90_ among genotypes (genotype-by-assay-environment interaction: corrected *P* > 0.05 for all antibiotics). Furthermore, we found no evidence that average IC_90_ was higher for mutants tested in sympatric environments (selection environment = assay environment) than in allopatric environments (selection environment ≠ assay environment), as would be the case if the mutations we detected conferred bigger increases in resistance in the abiotic conditions they were selected in (33, 34) (difference between sympatric and allopatric combinations: corrected *P* > 0.05 for all selection antibiotics). Thus, variation across assay environments of the relative changes in resistance (measured as IC_90_) conferred by different resistance mutations did not explain why we isolated different mutants in different conditions (Fig. 1).

10.1128/mSystems.01055-21.4 FIG S4 Resistance of mutants to the antibiotic they were isolated against (selection antibiotic), measured in four sets of abiotic conditions (assay environment). Each panel shows mutants selected for resistance to the drug indicated for the panel, with the resistance to this selection drug as IC_90_, relative to the ancestor in the same environment (Fig. S3). Mutants are grouped and colored according to the gene or gene set that was mutated, with the thick lines showing the mean IC_90_ across all strains with the same genotype. Where there were multiple mutants with the same genotype, thin lines show the antibiotic free growth for the individual mutants. The *y* axis is log transformed, and the scale varies between panels. Download FIG S4, TIF file, 0.4 MB. Copyright © 2021 Allen et al. 2021 Allen et al. https://creativecommons.org/licenses/by/4.0/ This content is distributed under the terms of the Creative Commons Attribution 4.0 International license.

We next analyzed an alternative measure of resistance, growth of each mutant at the antibiotic concentration used during selection (GASC). Our rationale here was that the mutations most beneficial during our screen (and most likely to result in formation of viable colonies) are not necessarily the mutations that confer the largest increases in IC_90_. Therefore, GASC potentially provides additional information about why some types of mutants were associated with particular selection environments. GASC was calculated from the same dose-response curves as the IC_90_ and was positively correlated with IC_90_ across all mutants (correlation: τ = 0.508; *P* < 0.0001) (Fig. S5). For trimethoprim-resistant mutants, GASC was predicted by the interaction between mutated gene and assay environment (gene-by-assay-environment interaction: χ^2^_6_ = 33.4; corrected *P* < 0.001) (Fig. 3d), and the best-performing individual mutant (thin lines in Fig. 3d) varied among different assay conditions. Trimethoprim-resistant mutants also showed evidence of matching between mutants and their selection environments, in that GASC was higher in sympatric than allopatric combinations (effect of sympatry: β = 0.0818; standard error [SE] = 0.0234; χ^2^_1_ = 11.8; corrected *P* < 0.01). For cefuroxime-resistant mutants, GASC varied significantly among sets of mutants selected under different abiotic conditions, with bile-selected mutants performing best (effect of selection environment: χ^2^_3_ = 11.3; corrected *P* < 0.05) (Fig. 3a). Thus, addition of bile biased our screen toward a relatively narrow set of mutants that grew well at the cefuroxime concentration used during selection (in particular, *ftsI* mutants) (Fig. 1). Consistent with this, we observed resistant colonies in our mutant screen in fewer replicate populations exposed to bile plus cefuroxime than other cefuroxime selection environments (Fig. S6). Despite this, the genotype-by-assay-environment interaction for cefuroxime-resistant mutants was not significant after accounting for multiple testing (χ^2^_9_ = 15.4; corrected *P* > 0.1). Note that *ftsI* mutants also had relatively high IC_90_ on average (Fig. S4), although this did not translate to significant variation of mean IC_90_ with selection environment, as it did for GASC. Finally, for the relatively small number of chloramphenicol-resistant mutants we tested, we also observed an interaction between mutated gene and assay environment for GASC (Fig. S2c). In summary, analyzing GASC revealed evidence that some selection environments favored particular types of resistance mutations.

10.1128/mSystems.01055-21.5 FIG S5 Correlation between GASC and IC_90_ for 48 mutants, each measured against the relevant selection drug in each of the four assay environments. Each point is from an independent dose-response assay, with IC_90_ and GASC calculated from the same data. The different measures are transformed according to the way that the variables are input into the relevant models. These measures are highly correlated (τ = 0.508; *P* < 0.0001). Download FIG S5, TIF file, 0.1 MB. Copyright © 2021 Allen et al. 2021 Allen et al. https://creativecommons.org/licenses/by/4.0/ This content is distributed under the terms of the Creative Commons Attribution 4.0 International license.

10.1128/mSystems.01055-21.6 FIG S6 Number of independent wells (of 92) which had resistant colonies after plating E. coli with different drugs in different environments. The number of populations in which resistance was seen (one or more resistant colonies) varied with the combination of antibiotic and selection environment (binomial glm of number of independent wells with resistance: antibiotic by environment interaction, χ^2^_12_ = 160; *P* < 0.0001). The dashed line shows the maximum number of independent mutants (i.e., 92). Download FIG S6, TIF file, 0.1 MB. Copyright © 2021 Allen et al. 2021 Allen et al. https://creativecommons.org/licenses/by/4.0/ This content is distributed under the terms of the Creative Commons Attribution 4.0 International license.

### Antibiotic-free growth depends on resistance mechanism and local abiotic conditions.

Antibiotic resistance is often associated with a fitness cost in terms of impaired growth in the absence of antibiotics, and variation of this cost is a key driver of the long-term persistence of resistance (16, 35). In our mutant selection experiment, bacteria were grown in the absence of antibiotics prior to plating. Because plating was done at antibiotic concentrations that fully inhibited growth of the ancestral strain, we expect resistance mutations that we detected to have arisen predominantly during this first (antibiotic-free) phase (Fig. S1), rather than on the agar plate. Therefore, variable costs of resistance across selection environments could potentially help to explain why we found different sets of mutants in different selection environments. We investigated this by quantifying the growth of each mutant in the absence of antibiotics, using the data set used to calculate the IC_90_s (see Materials and Methods). Relative to the ancestral strain in the absence of antibiotics (mean final optical density ± standard deviation, 0.898 ± 0.104), most types of resistant mutants showed evidence of growth costs, with 29 of the 57 mutants showing at least a 20% reduction in mean growth relative to the ancestor in one or more environment. However, this varied depending on the selection antibiotic (cefuroxime, 0.876 ± 0.194; gentamicin, 0.599 ± 0.308; streptomycin, 0.488 ± 0.258; trimethoprim, 0.790 ± 0.168) (Fig. 4).

For all selection antibiotics, mean antibiotic-free growth varied depending on which gene was mutated (Fig. 4, effect of mutated gene on antibiotic-free growth: cefuroxime-selected mutants, *F*_4,16_ = 107, corrected *P* < 0.001; gentamicin, *F*_5,12_ = 23.0, corrected *P* < 0.001; streptomycin, *F*_6,10_ = 15.5, corrected *P* < 0.001; trimethoprim, *F*_3,18_ = 358, *P* < 0.001). For example, streptomycin-resistant mutants with mutations in *atpG* grew relatively poorly (Fig. 4c) (difference from ancestor: β = −0.66, *t*_10_ = 5.67, *P* < 0.001), as did trimethoprim-resistant mutants with *phoPQ* mutations (Fig. 4d) (difference from ancestor: β = −0.20, *t*_15_ = 6.84, *P* < 0.001). For all selection antibiotics, variation of antibiotic-free growth among different genotypes depended on the local abiotic conditions (genotype-by-assay-environment interaction: cefuroxime, χ^2^_9_ = 23.8, corrected *P* < 0.05; gentamicin, χ^2^_12_ = 27.4, corrected *P* < 0.05; streptomycin, χ^2^_15_ = 33.4, corrected *P* < 0.05; and trimethoprim, χ^2^_6_ = 52.0, corrected *P* < 0.001). Despite this, we did not find any evidence of matching between mutants and their selection environments in terms of antibiotic-free growth (sympatric versus allopatric contrast: *P* > 0.05 for all selection antibiotics). In summary, local abiotic conditions modified the costs of resistance for our genotypes in the absence of antibiotics, but this did not explain the distribution of genotypes across selection environments in our mutant screen.

## DISCUSSION

Our findings have important implications for research that aims to exploit collateral sensitivity in novel treatment approaches. For example, among our trimethoprim-resistant mutants, those selected at high temperatures showed the greatest collateral sensitivity to nitrofurantoin. Thus, trimethoprim treatment of poultry (36), with a higher body temperature, could potentially select for mutants that are collaterally sensitive to nitrofurantoin, but this may be less likely in other environments. Studies of collateral sensitivity will therefore be most relevant when they account for environmental variation, for example by focusing on resistant mutants that arise under conditions similar to those during infection, or testing explicitly for variation across abiotic conditions (as done here). This will ensure that collateral effects of mutations specific to the infection environment are not overlooked, and that collaterally sensitive mutants specific to the lab environment are not given undue attention. Note that our study included only chromosomal mutants derived from a single laboratory strain, allowing us to study the mutants in a well-characterized genetic background. Nevertheless, some of the resistance mechanisms we identified are known to be important in natural and clinical populations, such as mutations in genes for efflux pumps (*acr*) (37), global regulators (*marR* and *phoPQ*) (38, 40) and specific antibiotic targets (*ftsI* and *rpsL*) (41–43), suggesting that our findings are relevant beyond laboratory studies. A key question for future work is whether collateral effects of resistance encoded on plasmids (44, 45), which is common in clinics, show sensitivity similar to that under abiotic conditions as we saw here. We speculate that this is likely, because plasmids can carry accessory genes with fitness effects that are strongly affected by the abiotic environment (46, 47). In support, a recent study suggested that plasmid carriage can induce collateral sensitivity in E. coli (48).

Some past studies have looked at collateral sensitivity in clinical isolates (10, 49, 50), but these studies each measured collateral sensitivity in a single lab environment. Our results suggest that this may risk overlooking collateral sensitivity interactions that may be important in the infection environment. For example, if we had tested *cpxA* mutants only in a single environment at 37°C, we would have observed only that they mediate cross-resistance between gentamicin and cefuroxime (51, 52). This would miss the collateral sensitivity of these mutants at higher temperature (Fig. 2b), a potentially interesting target for collateral sensitivity in E. coli infecting hosts with higher body temperatures, such as poultry (25). More generally, variable collateral effects as we observed here are relevant for understanding the evolution of antibiotic resistance across different conditions or environmental compartments (53) or in infections that start in one location, condition, or host before spreading to others (54, 55). The changing expression of collateral effects with abiotic conditions therefore represents an important variable which should be considered if we are to design robust treatment regimens around collateral sensitivity. Of course, our experiments were still restricted to simplified lab conditions. Our aim here was to demonstrate that even relatively minor (but still relevant to nature) manipulations of the abiotic environment can modify collateral sensitivity interactions. Our finding that such effects are strong suggests other differences between complex within-host or natural environments and *in vitro* screening conditions are likely to modify expression of collateral resistance even further.

It is worth noting that the method we used to generate resistant mutants differs from some other studies on collateral sensitivity (5, 6), where bacteria were exposed to sub-MICs or increasing antibiotic concentrations over multiple growth cycles. In our approach, as in a conventional fluctuation assay screening for antibiotic resistant mutants (56), we grew the ancestral strain in the absence of antibiotics to generate diversity, before treating with inhibitory concentrations of antibiotics to select for resistance to otherwise inhibitory concentrations. Thus, resistance emerges via selection at a high concentration for preexisting resistant variants (that arose prior to antibiotic exposure), rather than gradual exposure to increasing antibiotic concentrations that progressively increase selection for resistance and decrease population growth of the sensitive ancestral strain (57). We identified many mutations (e.g., in *hem* genes) that have been found in previous studies (5, 10). The method we used also parallels the course of many infections, as an infection is established in the absence of antibiotics and then a dose of antibiotics designed to fully inhibit the infection is used on the diverse population, either wiping it out or strongly selecting for any resistant subpopulation that is present (58, 59). Nevertheless, such single-step screens may select for a subset of all possible pathways to resistance, for example if there are multimutational pathways that emerge only upon gradual exposure (60).

In summary, we found that local abiotic conditions modified collateral sensitivity interactions by two principal mechanisms. First, antibiotic treatment can select for different genetic pathways to resistance depending on the local abiotic environment (Fig. 1). This was the case here for the selection antibiotics cefuroxime and streptomycin, after correcting for multiple testing (under less conservative criteria, this trend is more pervasive across antibiotic treatments). This can in turn alter the average strength of collateral sensitivity to other antibiotics (Fig. 2), as we observed with trimethoprim-resistant mutants, which were variably collaterally sensitive to nitrofurantoin depending on the abiotic environment they were selected in. Second, for individual mutants, expression of collateral sensitivity or cross-resistance can depend strongly on local abiotic conditions. For example, we found that gentamicin-resistant *cpxA* mutants were cross resistant or collaterally sensitive to cefuroxime depending on the assay environment. This is consistent with a more general trend that the phenotypic effects of antibiotic resistance mechanisms are highly sensitive to genotype-by-environment interactions (16–18, 61). Critically, this suggests that for some antibiotic combinations, the effectiveness of the second antibiotic against bacteria that have evolved resistance to the first antibiotic, and consequently selection on resistance to both antibiotics, will depend on the abiotic environment. While collateral sensitivity still holds great promise to prolong the effectiveness of available treatments, we suggest that doing so will be most effective if we account for local abiotic conditions.

## MATERIALS AND METHODS

### Organisms and growth conditions.

We used Escherichia coli K-12 MG1655 as the ancestral organism, grown at 37°C in static 100-μl cultures in 96-well microplates unless otherwise stated. The medium used was based on lysogeny broth (LB; Sigma-Aldrich) with additions to create variations in environmental conditions for basal, pH, and bile media. Basal medium (the base condition) is LB buffered at pH 7.0 with 0.1 M sodium hydrogen phosphate (Na_2_HPO_4_ and NaH_2_PO_4_). pH medium (acidic pH) is LB buffered at pH 6.5 with 0.1 M sodium hydrogen phosphate. Bile medium is LB with the addition of 0.5 g/liter of bile salts and buffered at pH 7.0 with 0.1 M sodium hydrogen phosphate. The temperature treatment uses the basal medium but is incubated at 42°C. When these conditions were on solid media (i.e., to select for resistant colonies), we instead used LB agar (Sigma-Aldrich), but other temperature and medium additions were the same. For overnight culture prior to assay, we incubated at 37°C in diluted LB (LB-water, 1:2).

### Mutant isolation.

We screened for mutants resistant to each selection antibiotic in each selection environment by first making 460 cultures of the ancestral E. coli strain for each of the 4 selection environments (1,840 cultures total, each inoculated with a small number of cells via dilution from a single frozen stock), incubated for 20 h in the absence of antibiotics. Each entire culture was then transferred to a well of a 24-well plate containing 1 ml of agar corresponding to the same selection environment as the prior liquid culture plus one of five antibiotics at selection concentration (cefuroxime [selection concentration = 6 μg mL^−1^], chloramphenicol [6 μg mL^−1^], gentamicin [24 μg mL^−1^], streptomycin [72 μg mL^−1^], and trimethoprim [0.5 μg mL^−1^]). These selection concentrations were approximately equal to MICs for the ancestor. This meant the ancestral strain was effectively inhibited, but mutants with even moderately increased resistance could grow. A higher selection concentration was used for the aminoglycosides (gentamicin, 48 μg mL^−1^; streptomycin, 144 μg mL^−1^) in the pH medium because of the reduced efficacy against the ancestral strain in this environment (62). The agar with bacterial culture was incubated for 48 h at 37 or 42°C. This protocol was used to screen 92 independent E. coli populations (each plated from a separate overnight culture grown in the absence of antibiotics) for each combination of 4 selection environments and 5 selection antibiotics. After incubation, each agar well was checked for the appearance of resistant colonies (the number of populations that produced at least one colony is shown in Fig. S6). Up to 6 colonies for each antibiotic-by-environment combination were picked from 6 randomly selected, independent agar wells (populations). These colonies were grown in LB without antibiotics (so as not to further select for resistance) for 3 h before glycerol was added to 25% of the final volume and mutants were frozen at −80°C. Mutant isolation in the basal, pH, and temperature environments was done in the same temporal block, and the bile environment in a separate block. These frozen stocks were used to inoculate cultures in LB without antibiotics to extract DNA for sequencing. For a minority of mutants, we were unable to consistently revive the frozen stock for library preparation for sequencing and/or for phenotyping. These were excluded from the relevant assays and analysis (see Table S2 for details).

10.1128/mSystems.01055-21.9 TABLE S2 Mutants used for phenotypic models. This table shows the 57 mutants which were used in the phenotypic models. For these mutants, we were able to identify mutations and to obtain replicate measures for one or more phenotypes of interest in all four abiotic environments. The “Sample Code” column shows which strain the information corresponds to (as in Table S1). “Selection drug” and “Selection environment” columns reflect the conditions each mutant was selected in. The “Gene Set affected” column shows the mutations in the isolate; where more than one gene set was mutated in a single mutant, only one gene set could be used for this variable to group the mutants based on genotype (Table S1). The final four columns show the number of data points used in the models for each of the four phenotypes. These are written as a string of four numbers, corresponding to the number of replicate measures for the relevant phenotype in basal, bile, pH, and temperature assay environments, respectively. “None” indicates that we did not have enough replication in one or more assay environments, so this mutant was not included in the model for this phenotype but was included in the models for other phenotypes. Note that the number of data points for growth in the absence of antibiotics is higher, as we were able to use the data from the dose-response curves of both the selection and paired drugs for each isolate, resulting in twice as many possible estimates. Download Table S2, DOCX file, 0.04 MB. Copyright © 2021 Allen et al. 2021 Allen et al. https://creativecommons.org/licenses/by/4.0/ This content is distributed under the terms of the Creative Commons Attribution 4.0 International license.

### Genome sequencing and bioinformatics.

Genomic DNA from 110 mutants plus the ancestral strain was extracted with the Genomic-tip 20/G kit (catalog no. 10223; Qiagen) according to the manufacturer’s instructions. Libraries were produced using the Illumina Nextera XT kit. Sequencing was performed on the Illumina HiSeq 4000 platform with 150-bp paired-end reads at the Functional Genomic Center, Zürich, Switzerland (Fig. 1). Reads were trimmed using Trimmomatic (63) and then analyzed using the breseq pipeline in consensus mode relative to the K-12 MG1655 reference, taking into account mutations present in our ancestral strain (64, 65). For each mutant, we then identified which genes were affected by mutations due to single nucleotide polymorphisms (SNPs) or insertions or deletions in the coding sequence or the promoter region of the gene (identified by breseq or in rare cases by manual curation) (Table S1) relative to the ancestral sequence. Four synonymous SNPs are shown in Fig. 1 but were excluded from our analysis. Several strains did not have mutations identified by breseq in consensus mode and were therefore not used for further analysis. Once we had completed these filtering steps, we gained full genotypic information for 84 strains.

For plotting and analysis, we accounted for differences both among individual mutants, and among genes or gene sets that were mutated in multiple individual mutants. For example, there were multiple mutants with mutations in *ftsI* (Fig. 1). A gene set here is a group of related genes (Table S1), including genes from the same operon (e.g., *phoQP*) or having very closely related functions (e.g., all *rib* genes). In one mutant, we found a large deletion affecting multiple genes, including *hemB*, which was also altered in some other mutants; this mutation was designated as affecting *hemB*. This list of genes and gene sets was used in PERMANOVA (see “Statistics”). Sixteen mutants had multiple mutations affecting multiple genes (Table S1); 11 of these mutants were included in the phenotypic analysis. In such cases, if one of the mutated genes was also mutated in other mutants, we categorized the strain according to this gene (e.g., CFR5Basal, categorized as *marR* because the other mutated gene in this mutant, *asmA*, was not mutated in any other mutant and because *marR* has a known role in resistance); in the other such cases, we categorized each mutant according to mutated genes known to be involved in resistance to the selection antibiotic (e.g., GEN4pH categorized as *nuoG*, because *nuoG* has been associated with resistance previously [5]). The choice of gene used for categorization and labeling did not affect the outcome of our analyses in these latter cases.

### Measuring resistance to selection antibiotics and other antibiotics.

We selected 75 strains with genotypic information to phenotype (and the ancestor), excluding mutants where very similar genotypes were already represented. Resistance of all mutants and the ancestral strain was quantified using broth dilution. For each antibiotic, we assayed each combination of strain, assay environment, and antibiotic concentration in four biological replicates, each in a separate temporal block. In each block of assays, we used a frozen master plate containing all strains organized in one of three randomized layouts (blocks 1, 2, 3, and 4 used layouts 1, 2, 3, and 1) to inoculate a single preculture plate (LB-water, 1:2). We then incubated the preculture plate for 3 h before using it to inoculate all the overnight plates (for every assay culture, we grew a separate overnight culture). Overnight cultures were then used to inoculate the assay plates using a pin replicator. Each mutant was tested against the relevant selection and paired antibiotics, and the ancestor was tested against all antibiotics, each at 8 concentrations (including zero) in each assay environment. After culturing assay plates for 20 h, we agitated the plates to resuspend bacteria and then measured the biomass of bacteria by optical density at 600 nm (OD_600_) using a spectrophotometer (Infinite 200 Pro; Tecan Trading AG, Switzerland). Due to the time taken to read 64 plates, incubation and plate reading was staggered and the order was randomized. Some mutants failed to regrow during overnight incubation, resulting in some assay wells not being inoculated. To filter out these false negatives, we excluded OD_600_ scores from assay plates that were <0.03, but only if the OD_600_ in the overnight well (prior to inoculation of the assay well) was also <0.03 (both after subtracting blanks).

### Calculation of summary phenotypes from dose-response data.

For each mutant strain, we calculated four phenotypes: (i) 90% inhibitory concentration (IC_90_) of the selection antibiotic, (ii) growth at selection concentration (GASC) for the selection antibiotic, (iii) IC_90_ of the paired antibiotic, and (iv) growth in the absence of antibiotics. These phenotypes were calculated from the dose-response relationship data for the selection and paired antibiotics (for each replicate separately). We fitted a Hill function using nonlinear least-squares analysis in R (66), using the nlsLM function in the minpack.LM package (67): OD = (*A* × *k^n^*)/(*k^n^* + *C^n^*), where OD is the measured optical density and *C* is the antibiotic concentration. *A* is then the asymptote, *k* is the inflection point of the curve, and *n* is the Hill parameter controlling curve steepness. Thus, growth in the absence of antibiotics is equal to *A*, so for each combination of mutant and assay environment, antibiotic-free growth is the mean of the *A* parameters across all dose-response curves for this strain and environment. The IC_90_ for the selection and paired antibiotics can be calculated using the following formula, taking the parameters from the relevant fitted curve.

$${\text{IC}}_{90}=\;e^{\left[\ln\left(k\right)+\frac{\text{ln}(9)}{n}\right]}$$

Finally, GASC is the value of the Hill function when the antibiotic concentration equals the selection concentration.

For some strain-antibiotic-environment combinations, we could not robustly fit a Hill function to some or all replicates, for example if there was very little growth inhibition or if the dose response was strongly stepwise. In these cases, where possible, we calculated phenotypes independently from the fitting of dose response curves (as is often done in other studies [6]). We estimated growth in the absence of antibiotics from the OD measured after growth without antibiotics. We took the IC_90_ as the lowest tested concentration where growth was below 10% of growth in the absence of antibiotics. Finally, we took growth at selection concentration as the OD score at the selection concentration of the antibiotic or the predicted score at the selection concentration, assuming a linear relationship between growth and antibiotic concentration between the two measured concentrations on either side of the selection concentration. We used these fit-independent methods for a minority of cases (4.76% for antibiotic free growth, 2.48% for selection antibiotic IC_90_, 3.73% for paired-antibiotic IC_90_, and 4.76% for GASC). In all cases, there was a strong correlation between the fit-dependent and fit-independent measures (Fig. S7).

10.1128/mSystems.01055-21.7 FIG S7 Correlation between the 4 phenotypes measured when calculated using a method that requires a fitted Hill function (fit dependent) and a method that does not require a fitted Hill function (fit independent). Points represent values calculated from individual replicate dose-response measurements. Only points where both fit-dependent and fit-independent methods were calculated are plotted. The correlation measured by Pearson’s correlation coefficient is given at the bottom right of each panel; in all cases, this was significant (*P* < 0.001). The red lines indicate exact identity. Download FIG S7, TIF file, 0.2 MB. Copyright © 2021 Allen et al. 2021 Allen et al. https://creativecommons.org/licenses/by/4.0/ This content is distributed under the terms of the Creative Commons Attribution 4.0 International license.

### Statistics.

We treated the mutants selected for resistance against different antibiotics as independent data sets, due to the difficulty in comparing resistance across multiple antibiotics. This meant that we had only 5 independent mutants for chloramphenicol, which limited our ability to draw conclusions about this antibiotic. Therefore, we do not discuss the chloramphenicol-resistant mutants in the text, but they are included in the supplemental information for completeness (Fig. S2). In each data set, we took the four phenotypes of interest (see above) as response variables in separate models. In each model, the replicate measures for each phenotype came from independent dose response curves (fitted to data collected in different blocks). Mutants with insufficiently replicated data (<2 replicates in any of the four assay environments) for a given phenotype were excluded from the analysis for that phenotype, meaning that we had between 53 and 57 mutants for each of the 4 phenotypes (Table S2). We transformed IC_90_s by taking log_2_(IC_90_) relative to the mean of the ancestral strain (log_2_ transformed) measured in the same environment. This controlled for any effects of assay environment on antibiotic inhibition of the ancestral strain (Fig. S3) and normalized the data. GASC was square root transformed to fit the assumption of normality but was not relative to the ancestor, as the GASC was not significantly different from zero in the ancestor (as expected, given that we set selection concentrations close to the ancestral MIC). Growth in the absence of antibiotics already fitted the assumption of normality and was not analyzed relative to the ancestor, because ancestral growth did not vary significantly between assay environments (χ^2^_3_ = 5.11; *P* > 0.05).

For each of the 4 phenotypes across the 4 antibiotic data sets, we fitted two mixed effects models (Table S3). The fixed effects of model A were as follows: phenotype ∼ genotype + assay_environment + genotype: assay_environment. Those for model B were as follows: phenotype ∼ selection_environment + assay_environment + sympatry. In these models, genotype is based on the gene set mutated (so that mutants with different mutations in the same gene or gene set have the same genotype) (Table S1), and sympatry is a binary vector indicating whether selection environment is the same as assay environment. Model A was used to test whether genotypes varied in their average phenotypes (effect of genotype) and whether that variation depended on the local abiotic conditions (genotype-by-assay-environment interaction). To test the effect of selection environment, we used a separate model (model B), because genotype and selection environment were often confounded. Model B was used to test whether each phenotype varied on average among sets of mutants isolated from different selection environments (effect of selection environment) and for evidence of matching between mutants and their selection environments (higher average phenotypic scores in sympatric compared to allopatric combinations, where sympatry means that selection environment is the same as assay environment, and allopatry means that selection environment is not the same as assay environment; tested by the main effect of sympatry) (34).

10.1128/mSystems.01055-21.10 TABLE S3 Significance of terms from phenotypic models reported in the text. Models A and B were separately fitted to the different phenotypes. The models were independently simplified to the minimal models by dropping nonsignificant (α = 0.05) terms (not included in higher-order interactions), as described in more detail in Materials and Methods. Grey cells are significant at an α value of 0.05 and are thus kept in minimal models. For the fixed-effect terms, cells are black if terms were still considered significant after multiple-testing correction (sequential Bonferroni); this was tested only for fixed effects. Download Table S3, DOCX file, 0.03 MB. Copyright © 2021 Allen et al. 2021 Allen et al. https://creativecommons.org/licenses/by/4.0/ This content is distributed under the terms of the Creative Commons Attribution 4.0 International license.

In both models, we included a nested random effect of strain (individual mutant ID) on intercept [+(1|Strain) in the lmer function] to account for variation between strains (68). We also included a random effect of block nested within strain on the intercept [+(1|Strain:Block) in the lmer function], to account for variation between measures of the same strain in different blocks. To prevent overfitting, the variance explained by these random effects was tested using a likelihood ratio test (on the maximal model) and nonsignificant terms were dropped, potentially reducing to a fixed-effects model if both random effects were dropped (Table S3). After random effects were tested, we dropped nonsignificant fixed-effect terms to reach minimal models. For model simplification, both fixed-effect and random-effect terms were tested at an α value of 0.05.

Significance of terms in models is reported from minimal models by comparing models with or without the term of interest using an *F* test for fixed-effects models and a likelihood ratio test (χ^2^ statistic) for mixed models. When the main effect of genotype is involved in a significant higher-order interaction (genotype: assay_environment) the main effect cannot be dropped, so significance is instead reported with an *F* test (on a type III ANOVA), regardless of whether the model contains random effects. For mixed models, the approximate degrees of freedom are calculated using lmerTest (69). Although assay environment is included in both models, we report the significance of the main effect of assay environment from model B, where it does not have higher-order interactions and can always be tested using a likelihood ratio test.

To test whether the genes/gene sets that were mutated varied depending on the selection environment mutants were isolated from, we used a permutational ANOVA on the data for which genes/gene sets were affected by the mutations (Table S2, full genotype). This was performed using the adonis function in the vegan package (70).

For both the genotype and phenotype models, we tested 4 data sets (from four antibiotic combinations) for similar effects in parallel, therefore, when reporting the results of these tests, we give *P* values corrected for multiple testing using the Holm-Bonferroni method (sequential Bonferroni). These are reported in the text as corrected *P* values, and values below 0.05 are considered significant.

### Data availability.

All raw data are available on Dryad (https://doi.org/10.5061/dryad.6m905qg16). Genomic data are relative to the reference genome of Escherichia coli K-12 MG1655 and have been uploaded as SNP tables relative to this reference in the Dryad data file.
