# Supplementary figures and images for: Collateral Sensitivity Interactions between Antibiotics Depend on Local Abiotic Conditions
Source: mSystems. 2021 Nov 30;6(6):e01055-21. doi: 10.1128/mSystems.01055-21 (PMC8631318; doi:10.1128/mSystems.01055-21)

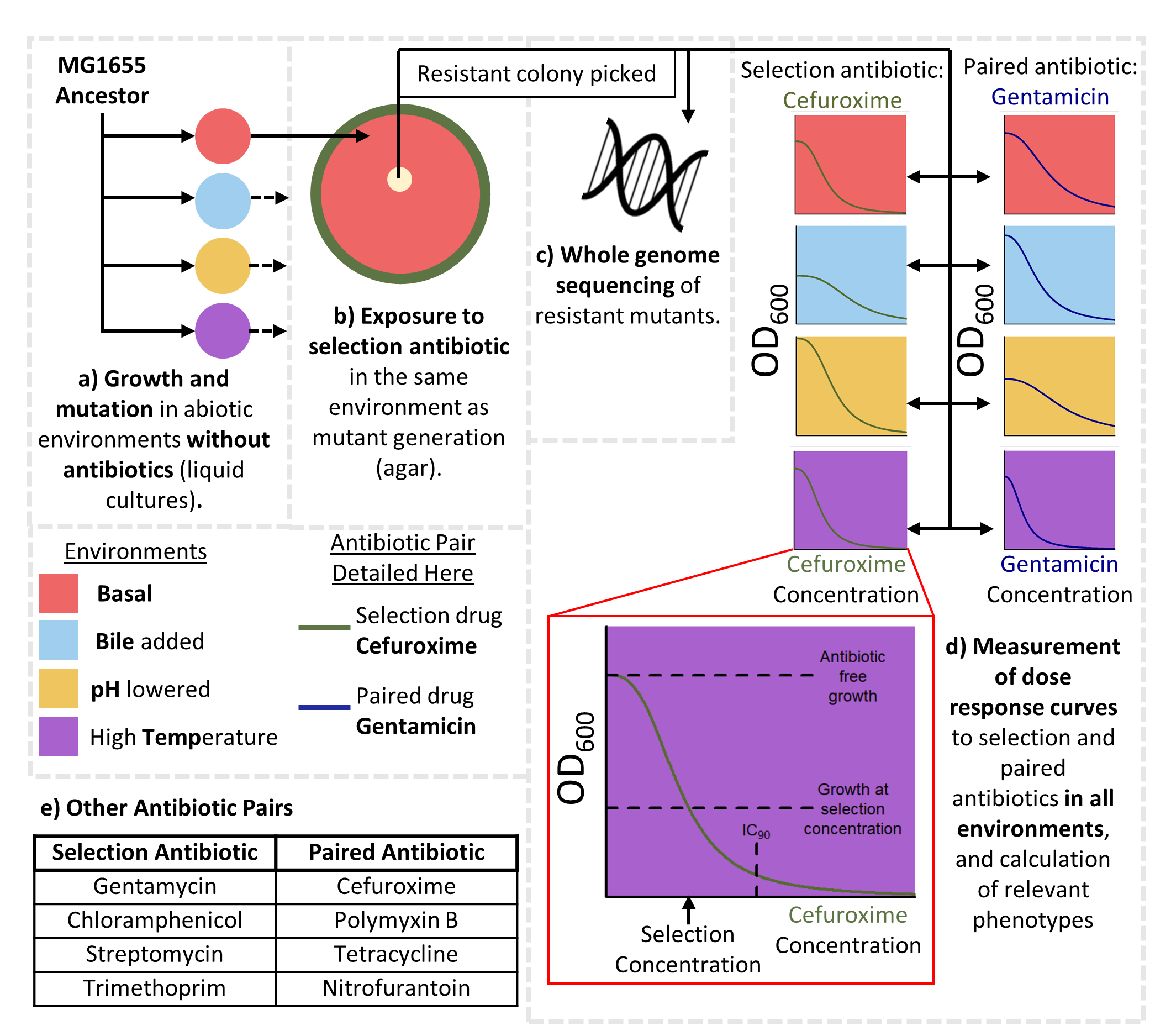

Supplement: FIG S1 [file msystems.01055-21-sf001.tif]

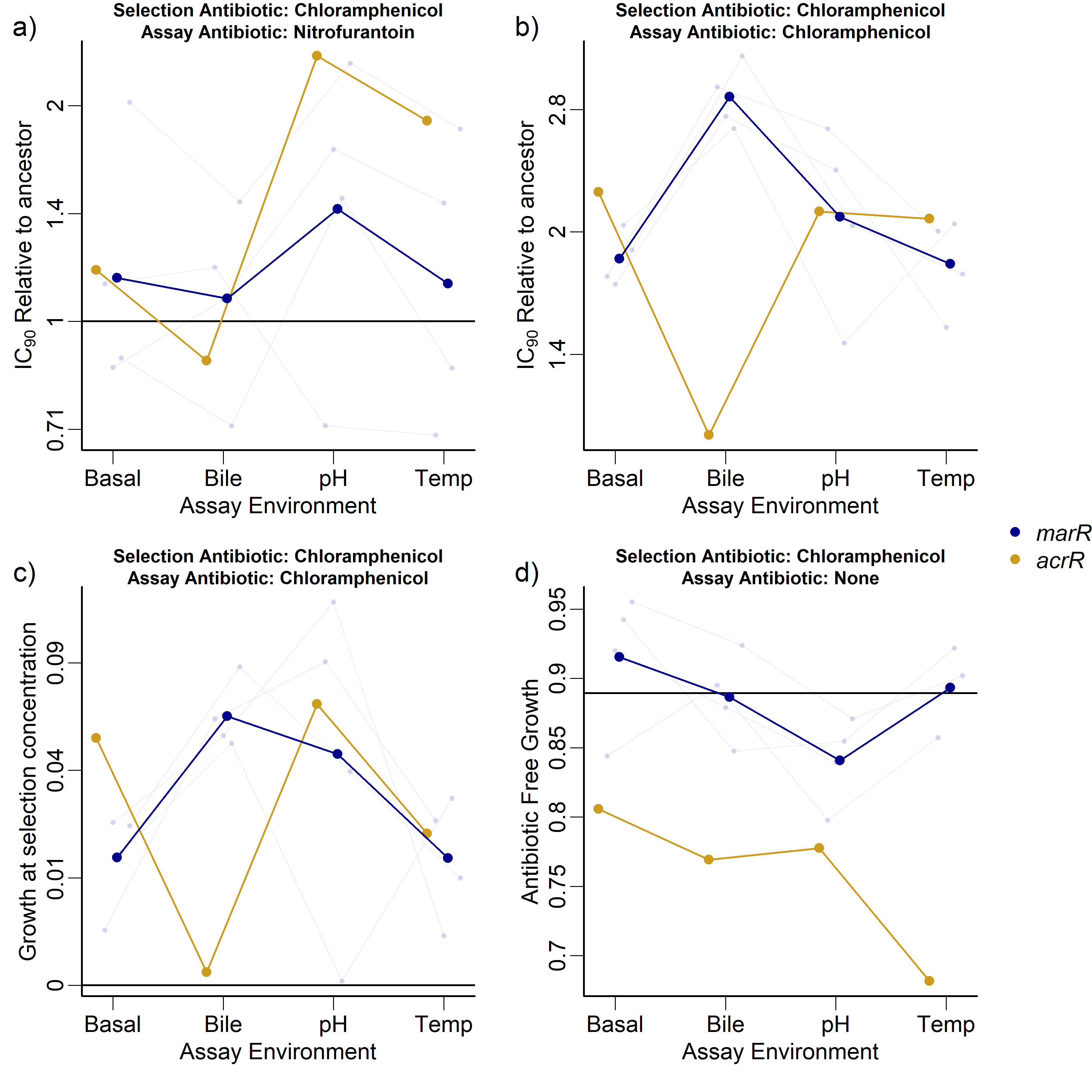

Supplement: FIG S2 [file msystems.01055-21-sf002.tif]

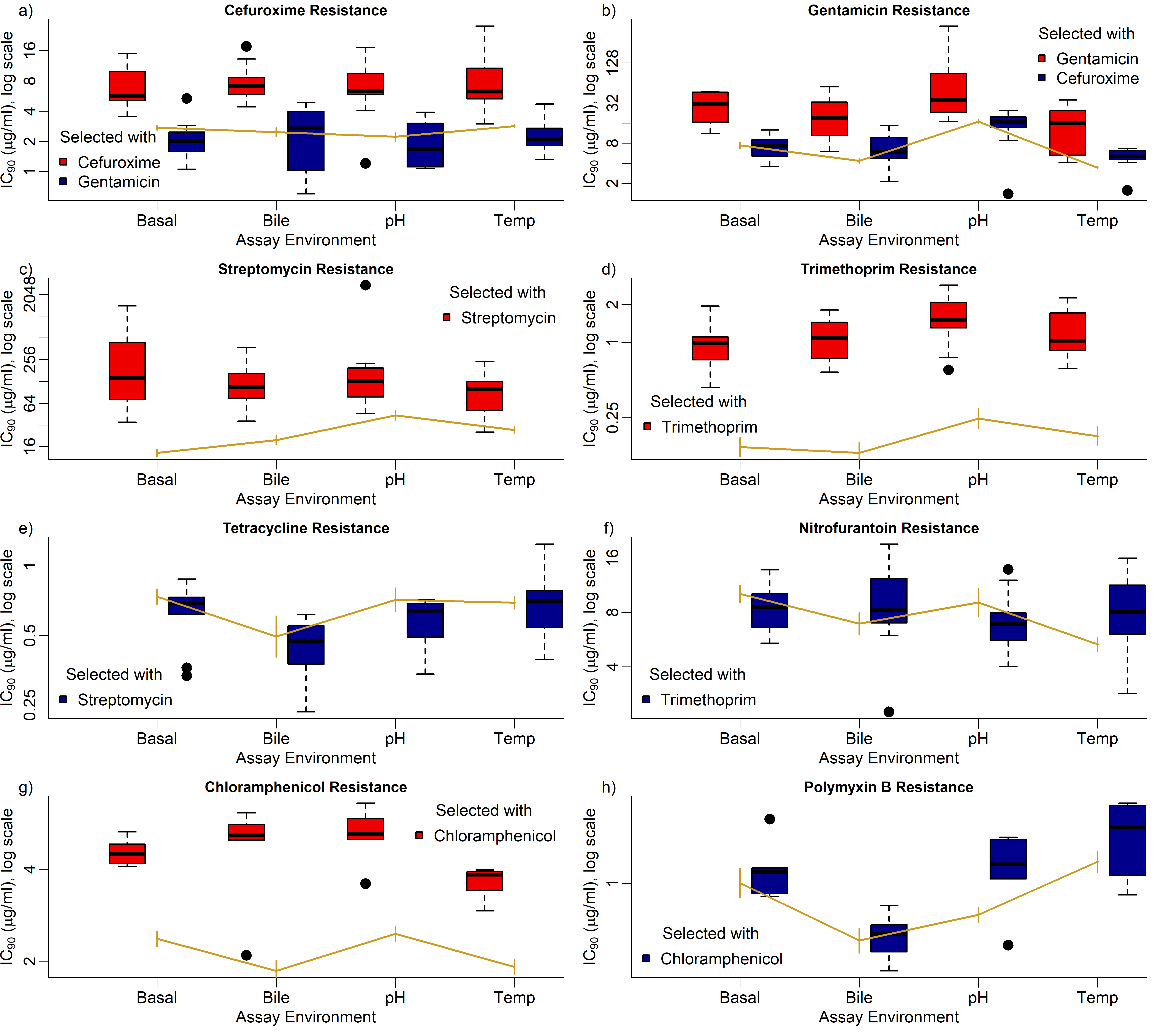

Supplement: FIG S3 [file msystems.01055-21-sf003.tif]

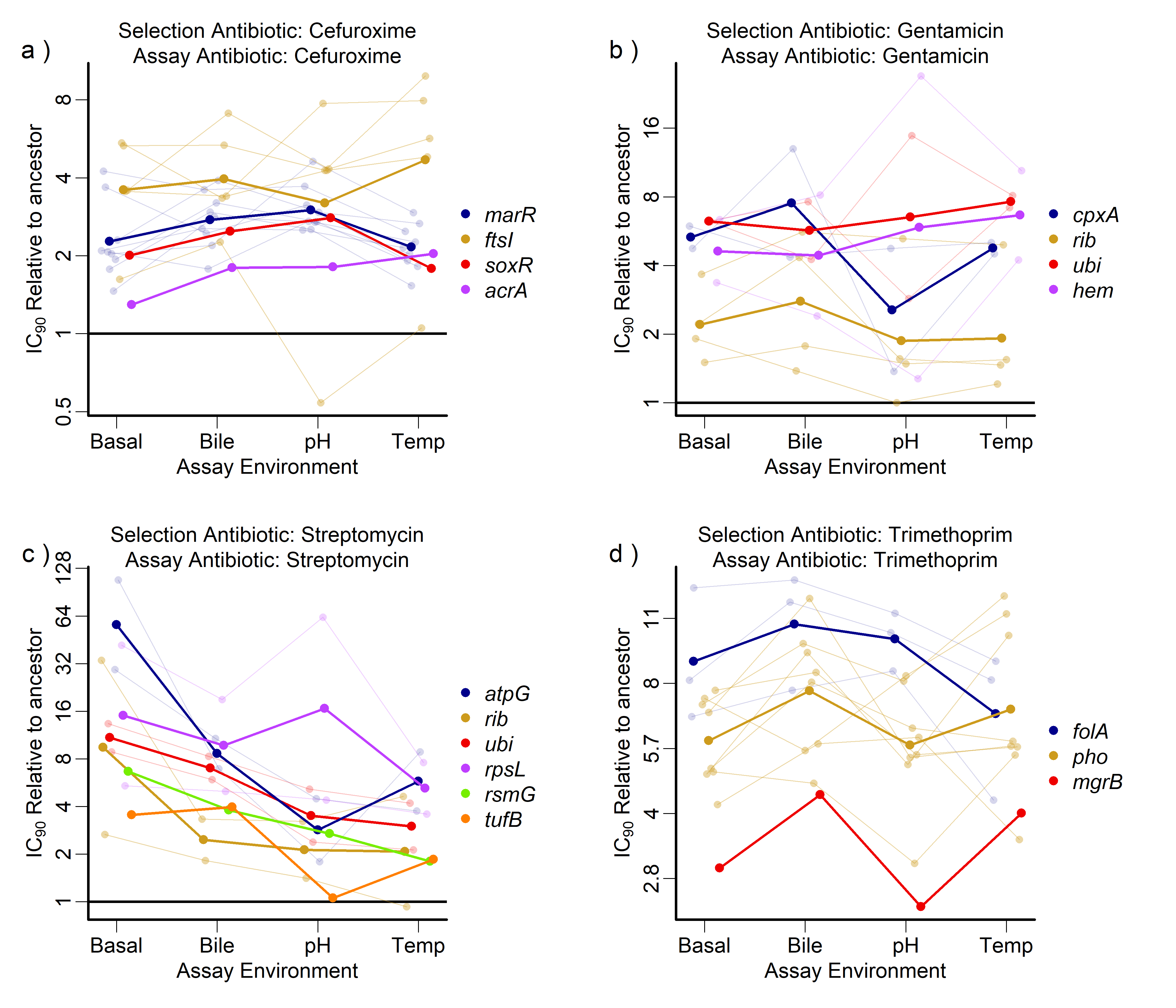

Supplement: FIG S4 [file msystems.01055-21-sf004.tif]

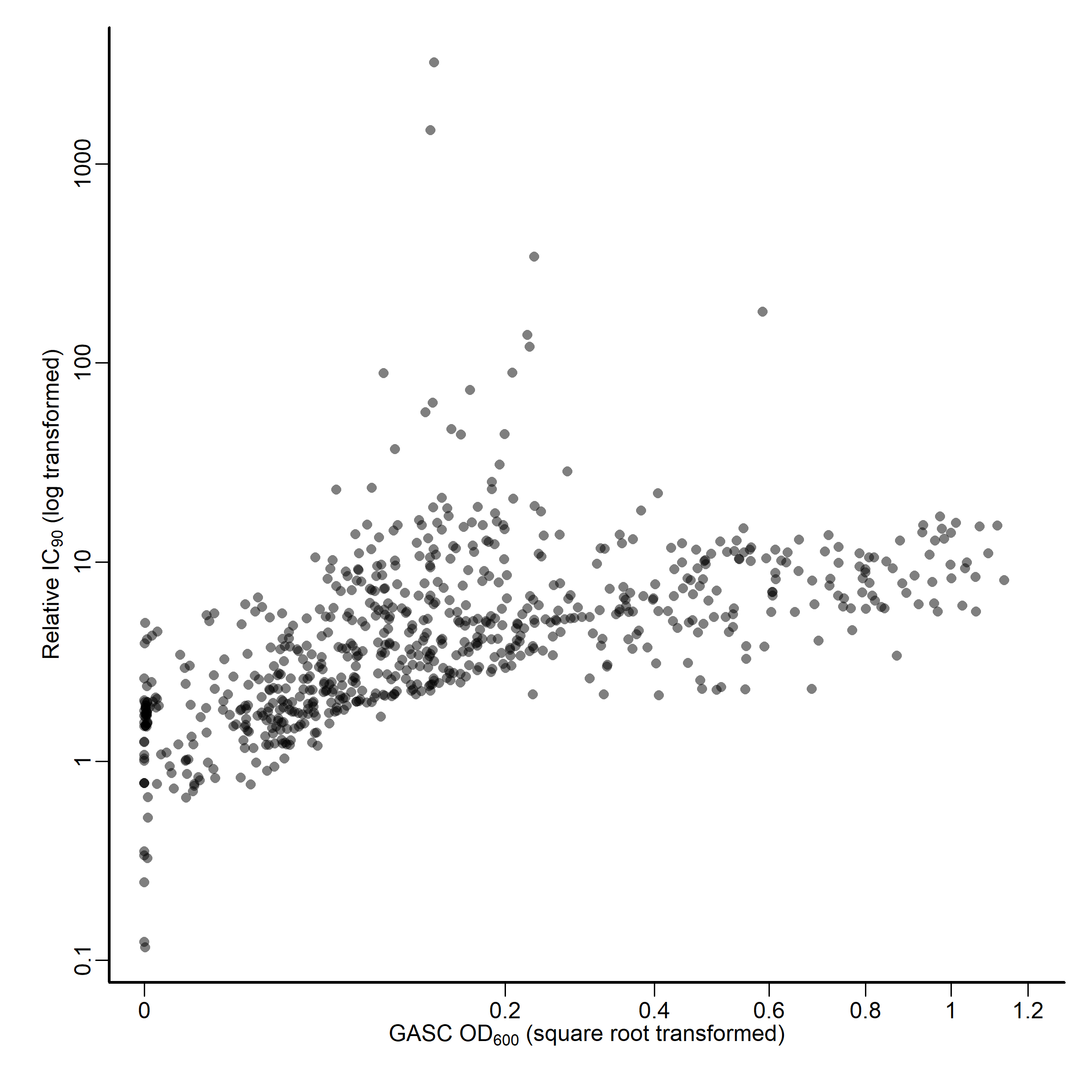

Supplement: FIG S5 [file msystems.01055-21-sf005.tif]

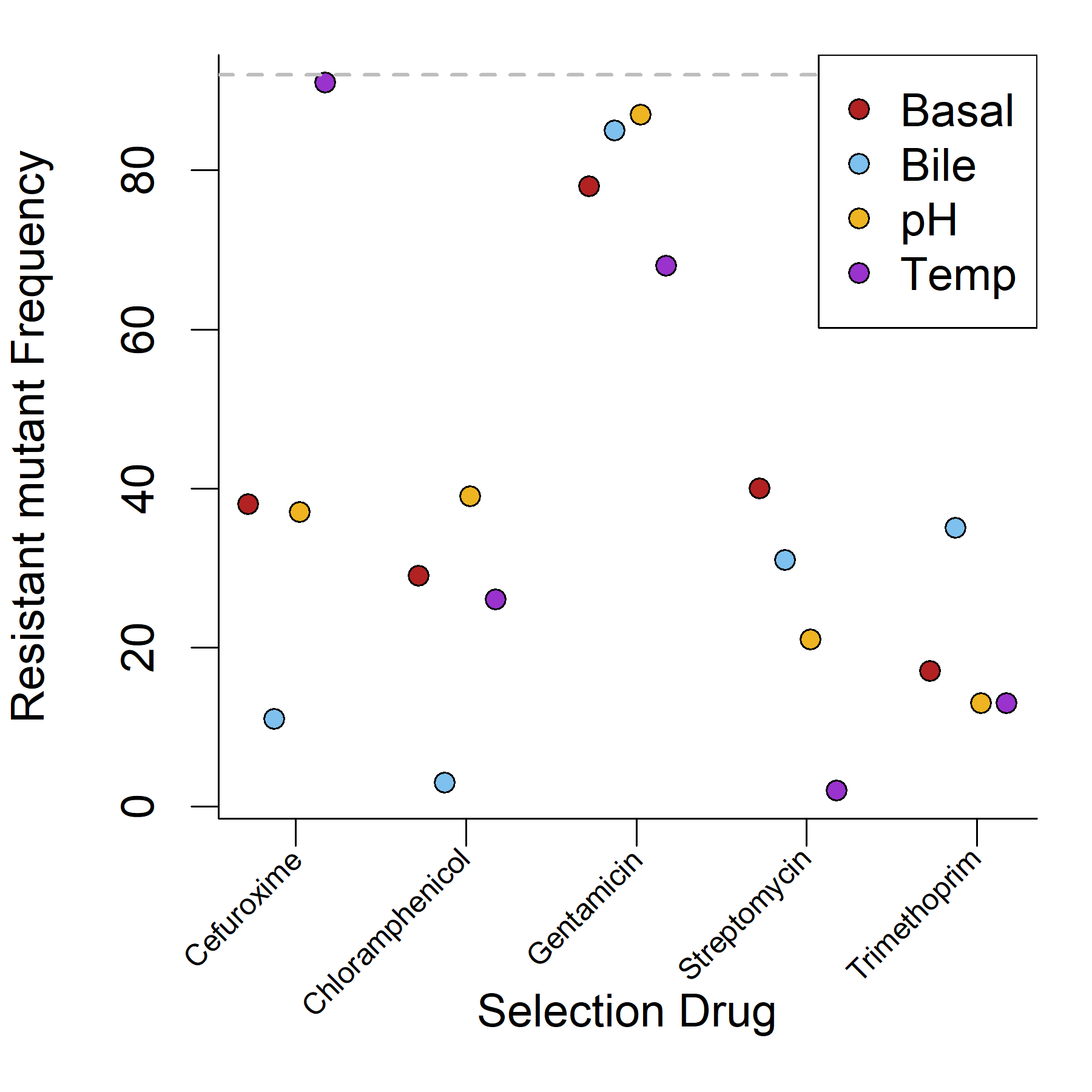

Supplement: FIG S6 [file msystems.01055-21-sf006.tif]

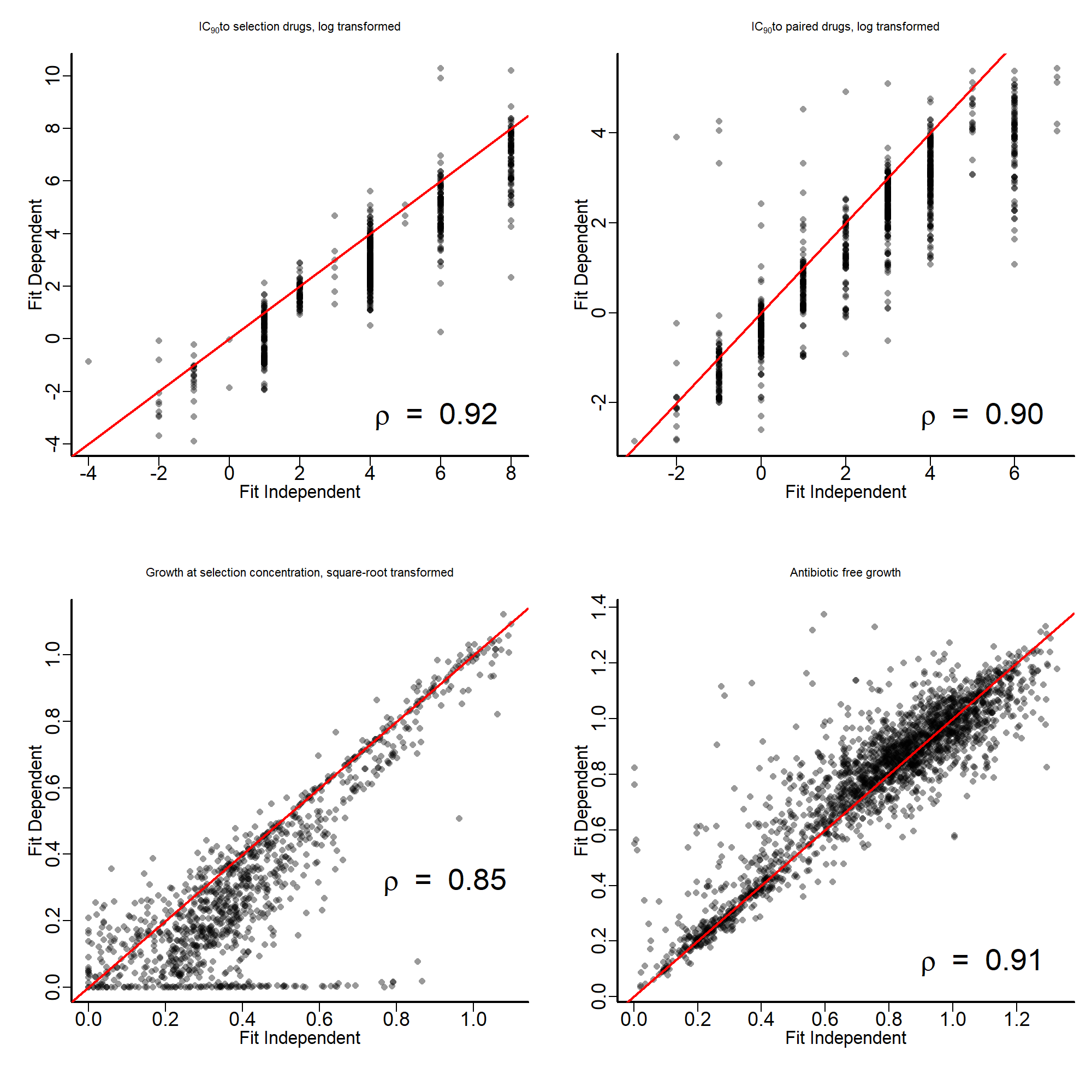

Supplement: FIG S7 [file msystems.01055-21-sf007.tif]
