# Supplementary material for: Collateral Sensitivity Interactions between Antibiotics Depend on Local Abiotic Conditions
Source: mSystems. 2021 Nov 30;6(6):e01055-21. doi: 10.1128/mSystems.01055-21 (PMC8631318; doi:10.1128/mSystems.01055-21)
Supplement: TABLE S1 [file msystems.01055-21-st001.docx]

| Sample Code | Genome position (bp) | Gene Name (bp in gene or from genes) | Gene Affected | Gene Set Affected | Details | Overall  average  coverage |
| --- | --- | --- | --- | --- | --- | --- |
| CFR1Basal | 1617358 | marR (214) | marR | mar | Nonsynonymous SNP (T->P) | 208 |
| CFR2Basal | 1617335 | marR (191) | marR | mar | 8bp insertion | 145 |
| CFR3Basal | 1617281 | marR (137) | marR | mar | Nonsynonymous SNP (L->P) | 133 |
| CFR4Basal | 1617263 | marR (119) | marR | mar | Nonsynonymous SNP (A->E) | 149 |
| CFR5Basal | 2136841 | yegH/asmA (+107/+167) | asmA^3^ | (asm) | Intergenic SNP | 126 |
| CFR5Basal | 1617229 | marR (85-234) | marR | mar | 150bp deletion | 126 |
| CFR6Basal | 1617125 | marC/marR (-192/-1) | marR^1^ | mar | 20bp deletion | 182 |
| CFR1Bile | 1617377 | marR (233) | marR | mar | Nonsynonymous SNP (L->R) | 205 |
| CFR2Bile | 92515 | ftsI (1103) | ftsI | fts | Nonsynonymous SNP (A->E) | 167 |
| CFR3Bile | 92650 | ftsI (1238) | ftsI | fts | Nonsynonymous SNP (A->V) | 134 |
| CFR4Bile | 92904 | ftsI (1492) | ftsI | fts | Nonsynonymous SNP (A->T) | 162 |
| CFR5Bile | 92823 | ftsI (1411) | ftsI | fts | Nonsynonymous SNP (M->V) | 196 |
| CFR5Bile | 3295752 | yraR (342) | yraR^2^ |  | Synonymous SNP | 196 |
| CFR6Bile | 92823 | ftsI (1411) | ftsI | fts | Nonsynonymous SNP (M->V) | 198 |
| CFR1pH | 1617558 | marR (414-415) | marR | mar | 2bp deletion | 145 |
| CFR2pH | 1617538 | marR (394-416) | marR | mar | 23bp deletion | 189 |
| CFR3pH | 4274368 | soxR (411) | soxR | sox | Nonsynonymous SNP (D->E) | 128 |
| CFR4pH | - | - | - | - | No mutations identified | 206 |
| CFR5pH | - | - | - | - | No mutations identified | 180 |
| CFR6pH | 1753774 | pykF (52) | pykF^3^ | (pyk) | Nonsynonymous SNP (E->K) | 116 |
| CFR6pH | 1617395 | marR (251) | marR | mar | Nonsynonymous SNP (V->E) | 116 |
| CFR1Temp | - | - | - | - | No mutations identified | 156 |
| CFR2Temp | - | - | - | - | No mutations identified | 181 |
| CFR3Temp | - | - | - | - | No mutations identified | 145 |
| CFR4Temp | - | - | - | - | No mutations identified | 158 |
| CFR5Temp | 485279 | acrR (295) | acrR | acr | Nonsynonymous SNP (S->P) | 168 |
| CFR6Temp | - | - | - | - | No mutations identified | 165 |
| CHL1Basal | - | - | - | - | No mutations identified | 177 |
| CHL2Basal | - | - | - | - | No mutations identified | 168 |
| CHL3Basal | - | - | - | - | No mutations identified | 184 |
| CHL4Basal | - | - | - | - | No mutations identified | 211 |
| CHL5Basal | - | - | - | - | No mutations identified | 160 |
| CHL6Basal | - | - | - | - | No mutations identified | 182 |
| CHL1Bile | 1617329 | marR (185) | marR | mar | 1bp deletion | 171 |
| CHL2Bile | 1617147 | marR (3) | marR | mar | Nonsynonymous SNP (M->V) | 174 |
| CHL3Bile | 1617493 | marR (349) | marR | mar | Nonsense SNP (Q->*) | 185 |
| CHL1pH | 2106183 | wzxB (651) | wzxB^2^ |  | Synonymous SNP | 212 |
| CHL1pH | 1617196 | marR (52-62) | marR | mar | 11bp deletion | 212 |
| CHL2pH | - | - | - | - | No mutations identified | 172 |
| CHL3pH | - | - | - | - | No mutations identified | 148 |
| CHL4pH | - | - | - | - | No mutations identified | 158 |
| CHL5pH | 484955 | acrA/acrR (-112/-30) | acrA | acr | Intergenic SNP | 136 |
| CHL6pH | - | - | - | - | No mutations identified | 121 |
| CHL1Temp | - | - | - | - | No mutations identified | 177 |
| CHL2Temp | - | - | - | - | No mutations identified | 151 |
| CHL3Temp | - | - | - | - | No mutations identified | 154 |
| CHL4Temp | - | - | - | - | No mutations identified | 166 |
| CHL5Temp | - | - | - | - | No mutations identified | 165 |
| CHL6Temp | - | - | - | - | No mutations identified | 160 |
| GEN1Basal | 3181941 | ribB/yqiC (-228/-141) | ribB^1^ | rib | IS186 (6bp) mobilisation | 133 |
| GEN2Basal | 4101418 | cpxA (42-47) | cpxA | cpx | 5bp deletion | 194 |
| GEN3Basal | 4101179 | cpxA (286) | cpxA | cpx | Nonsynonymous SNP (G->S) | 182 |
| GEN4Basal | 554648 | cysA (814) | cysS^3^ | (cys) | Nonsynonymous SNP (G->R) | 175 |
| GEN4Basal | 3469828 | fusA (175) | fusA | fus | Nonsynonymous SNP (R->C) | 175 |
| GEN5Basal | 3038938 | ygfZ (379-380) | ygfZ | ygf | 2bp deletion | 157 |
| GEN5Basal | 220871 | metQ (58) | metQ^2^ |  | Synonymous SNP | 157 |
| GEN6Basal | 3468174 | fusA (1829) | fusA | fus | Nonsynonymous SNP (P->L) | 186 |
| GEN1Bile | 3181747 | ribB/yqiC (-34/-340) | ribB | rib | Intergenic SNP | 156 |
| GEN1Bile | 3181941 | ribB/yqiC (-228/-141) | ribB^1^ | rib | IS186 (6bp) mobilisation | 156 |
| GEN2Bile | - | - | - | - | No mutations identified | 185 |
| GEN3Bile | 1208165 | tfaP (332-336) | tfaP^3^ | (tfa) | IS2 (5bp) mobilsation | 157 |
| GEN3Bile | 438354 | dxs (1048) | dxs | dxs | Nonsynonymous SNP (S->P) | 157 |
| GEN4Bile | 3050100 | ubiH (666) | ubiH | ubi | 2bp insertion | 175 |
| GEN5Bile | 3986654 | hemC (602) | hemC | hem | Nonsynonymous SNP (V->E) | 157 |
| GEN6Bile | 4016278 | ubiJ (166) | ubiJ | ubi | Nonsense SNP (Q->*) | 174 |
| GEN1pH | 4101096 | cpxA (274-369) | cpxA | cpx | 96bp deletion | 176 |
| GEN2pH | 3181941 | ribB/yqiC (-228/-141) | ribB^1^ | rib | IS186 (6bp) mobilisation | 191 |
| GEN3pH | 3050304 | ubiH (462) | ubiH | ubi | 1bp deletion | 147 |
| GEN4pH | 2397117 | nuoG (296) | nuoG | nuo | Nonsynonymous SNP (H->L) | 146 |
| GEN4pH | 2531630 | ptsI (318) | ptsI^3^ | (pts) | 1bp insertion | 146 |
| GEN5pH | 4101418 | cpxA (42-47) | cpxA | cpx | 6bp deletion | 117 |
| GEN6pH | 388344 | hemB (608) | hemB | hem | Nonsynonymous SNP (P->L) | 124 |
| GEN1Temp | 1522455 | yncE/ansP (+62/+51) | ansP^3^ | (ans) | Intergenic SNP | 174 |
| GEN1Temp | 3181941 | ribB/yqiC (-228/-141) | ribB^1^ | rib | IS186 (6bp) mobilisation | 174 |
| GEN2Temp | 3181941 | ribB/yqiC (-228/-141) | ribB^1^ | rib | IS186 (6bp) mobilisation | 157 |
| GEN4Temp | 3049693 | ubiH (1073) | ubiH | ubi | 1bp deletion | 167 |
| GEN5Temp | 174462 | hemL (421) | hemL | hem | Nonsynonymous SNP (C->R) | 162 |
| GEN6Temp | 434268 | ribE (398-409) | ribE | rib | 12bp deletion | 163 |
| STR1Basal | 22247 | ribF (841) | ribF | rib | Nonsynonymous SNP (R->C) | 203 |
| STR2Basal | 438114 | dxs (1288) | dxs | dxs | Nonsynonymous SNP (T->A) | 207 |
| STR3Basal | 694652 | ubiF (328-331) | ubiF | ubi | 4bp deletion | 135 |
| STR4Basal | 3181745 | ribB/yqiC (-32/-342) | ribB | rib | Intergenic SNP | 163 |
| STR4Basal | 3181941 | ribB/yqiC (-228/-141) | ribB^1^ | rib | IS186 (6bp) mobilisation | 163 |
| STR4Basal | 533272 | gcl (133) | gcl^3^ | (gcl) | Nonsynonymous SNP (H->N) | 163 |
| STR5Basal | 3181941 | ribB/yqiC (-228/-141) | ribB^1^ | rib | IS186 (6bp) mobilisation | 155 |
| STR5Basal | 1916040 | yebT (506) | yebT^3^ | (yeb) | Nonsynonymous SNP (D->A) | 155 |
| STR5Basal | 3181744 | ribB/yqiC (-31/-343) | ribB | rib | Intergenic SNP | 155 |
| STR6Basal | 3181941 | ribB/yqiC (-228/-141) | ribB^1^ | rib | IS186 (6bp) mobilisation | 202 |
| STR6Basal | 3181749 | ribB/yqiC (-36/-338) | ribB | rib | Intergenic SNP | 202 |
| STR1Bile | 3914040 | atpG (712-715) | atpG | atp | 4bp deletion | 165 |
| STR2Bile | 770530 | mngB/cydA (+695/-149) | cydA | cyd | IS5 (4bp) mobilisation | 147 |
| STR3Bile | 694520 | ubiF (196-199) | ubiF | ubi | IS5 (4bp) mobilisation | 156 |
| STR4Bile | 433606 | ribD (928) | ribD | rib | Nonsense SNP (Q->*) | 179 |
| STR5Bile | 4017322 | ubiB (608) | ubiB | ubi | Nonsynonymous SNP (E->G) | 176 |
| STR6Bile | 434262 | ribE (392-403) | ribE | rib | 12bp deletion | 202 |
| STR1pH | 3350004 | arcB (269) | arcB^3^ | (arc) | Nonsynonymous SNP (L->Q) | 156 |
| STR1pH | 3470785 | rpsL (256) | rpsL | rps | Nonsynonymous SNP (R->S) | 156 |
| STR2pH | 3920078 | rsmG (92) | rsmG | rsm | Nonsynonymous SNP (L->R) | 189 |
| STR3pH | 3470919 | rpsL (122) | rpsL | rps | Nonsynonymous SNP (T->I) | 168 |
| STR4pH | 3470811 | rpsL (230) | rpsL | rps | Nonsynonymous SNP (H->L) | 124 |
| STR5pH | 4172686 | tufB (254) | tufB | tuf | Nonsynonymous SNP (H->L) | 182 |
| STR6pH | 315708 | 78 genes | hemB^4^ | hem | 83492bp deletion | 186 |
| STR1Temp | 3914031 | atpG (724) | atpG | atp | 5bp insertion | 193 |
| TRM1Basal | - | - | - | - | No mutations identified | 158 |
| TRM2Basal | 50280 | folA (458) | folA | fol | Nonsynonymous SNP (F->S) | 184 |
| TRM3Basal | 1189100 | phoP (572) | phoP | pho | Nonsynonymous SNP (D->A) | 152 |
| TRM4Basal | 50280 | folA (458) | folA | fol | Nonsynonymous SNP (F->S) | 146 |
| TRM5Basal | 49765 | kefC/folA (+134/-58) | folA | fol | Intergenic SNP | 167 |
| TRM6Basal | 49910 | folA (88) | folA | fol | Nonsynonymous SNP (W->R) | 127 |
| TRM1Bile | 49912 | folA (90) | folA | fol | Nonsynonymous SNP (W->C) | 185 |
| TRM2Bile | 1189420 | phoP (252) | phoP | pho | Nonsynonymous SNP (W->C) | 143 |
| TRM3Bile | 1906692 | mgrB (100) | mgrB | mgr | 1bp deletion | 138 |
| TRM4Bile | 1188175 | phoQ (826) | phoQ | pho | Nonsynonymous SNP (T->P) | 134 |
| TRM5Bile | 1906837 | mgrB/yobH (-46/-110) | mgrB | mgr | IS5 (4bp) mobilisation | 143 |
| TRM6Bile | 800548 | ybhH (566) | ybhH^3^ | (ybh) | Nonsynonymous SNP (V->E) | 187 |
| TRM6Bile | 1189100 | phoP (572) | phoP | pho | Nonsynonymous SNP (D->A) | 187 |
| TRM2pH | - | - | - | - | No mutations identified | 153 |
| TRM3pH | 49903 | folA (81) | folA | fol | Nonsynonymous SNP (D->E) | 202 |
| TRM4pH | 1187850 | phoQ (1151) | phoQ | pho | Nonsynonymous SNP (G->D) | 156 |
| TRM5pH | 2931781 | fucP (300) | fucP^2^ |  | Synonymous SNP | 157 |
| TRM5pH | 1189428 | phoP (244) | phoP | pho | Nonsynonymous SNP (E->K) | 157 |
| TRM6pH | 49910 | folA (88) | folA | fol | Nonsynonymous SNP (W->G) | 145 |
| TRM1Temp | 1187850 | phoQ (1151) | phoQ | pho | Nonsynonymous SNP (G->V) | 195 |
| TRM2Temp | 1188381 | phoQ(620) | phoQ | pho | Nonsynonymous SNP (I->N) | 127 |
| TRM3Temp | 1188216 | phoQ (663-785) | phoQ | pho | 123bp deletion | 197 |
| TRM3Temp | 225600 | alaV/rrlH (+25/-159) | rrlH^3^ | (rrl) | Intergenic SNP | 197 |
| TRM4Temp | 50280 | folA (458) | folA | fol | Nonsynonymous SNP (F->S) | 181 |
| TRM5Temp | 3029475 | uacT (862) | uacT^3^ | (uac) | Nonsynonymous SNP (S->A) | 156 |
| TRM5Temp | 1187755 | phoQ (1246) | phoQ | pho | Nonsynonymous SNP (D->N) | 156 |
| TRM6Temp | 1189125 | phoP (547) | phoP | pho | Nonsynonymous SNP (E->K) | 188 |
