# Supplementary material for: Collateral Sensitivity Interactions between Antibiotics Depend on Local Abiotic Conditions
Source: mSystems. 2021 Nov 30;6(6):e01055-21. doi: 10.1128/mSystems.01055-21 (PMC8631318; doi:10.1128/mSystems.01055-21)
Supplement: TABLE S2 [file msystems.01055-21-st002.docx]

| Sample Code | Selection drug | Selection environment | Gene Set affected | Replicates included in analysis  Basal \| Bile \| pH \| Temp | | | |
| --- | --- | --- | --- | --- | --- | --- | --- |
|  |  |  |  | Paired IC_90_ | Selection IC_90_ | GASC | Antibiotic Free Growth |
| CFR1Basal | Cefuroxime | Basal | mar | 4\|4\|4\|4 | 4\|4\|4\|4 | 4\|4\|4\|4 | 8\|8\|8\|8 |
| CFR2Basal | Cefuroxime | Basal | mar | 4\|4\|4\|4 | 4\|4\|4\|4 | 4\|4\|4\|4 | 8\|8\|8\|8 |
| CFR3Basal | Cefuroxime | Basal | mar | 4\|4\|4\|4 | 4\|4\|4\|4 | 4\|4\|4\|4 | 8\|8\|8\|8 |
| CFR4Basal | Cefuroxime | Basal | mar | 4\|4\|4\|4 | 4\|4\|4\|4 | 4\|4\|4\|4 | 8\|8\|8\|8 |
| CFR5Basal | Cefuroxime | Basal | mar | 4\|4\|4\|4 | 4\|4\|4\|4 | 4\|4\|4\|4 | 8\|8\|8\|8 |
| CFR6Basal | Cefuroxime | Basal | mar | 4\|4\|4\|4 | 4\|4\|4\|4 | 4\|4\|4\|4 | 8\|8\|8\|8 |
| CFR2Bile | Cefuroxime | Bile | fts | 4\|3\|2\|3 | 3\|3\|3\|3 | 3\|3\|3\|3 | 7\|6\|6\|6 |
| CFR3Bile | Cefuroxime | Bile | fts | 4\|4\|4\|4 | 4\|4\|4\|4 | 4\|4\|4\|4 | 8\|8\|8\|8 |
| CFR4Bile | Cefuroxime | Bile | fts | 4\|4\|4\|4 | 4\|4\|4\|4 | 4\|4\|4\|4 | 8\|8\|8\|8 |
| CFR5Bile | Cefuroxime | Bile | fts | 4\|4\|4\|4 | 4\|4\|4\|4 | 4\|4\|4\|4 | 8\|8\|8\|8 |
| CFR6Bile | Cefuroxime | Bile | fts | 4\|4\|4\|4 | 4\|4\|4\|4 | 4\|4\|4\|4 | 8\|8\|8\|8 |
| CFR1pH | Cefuroxime | pH | mar | 4\|4\|4\|4 | 4\|4\|4\|4 | 4\|4\|4\|4 | 8\|8\|8\|8 |
| CFR2pH | Cefuroxime | pH | mar | 4\|4\|4\|4 | 4\|4\|4\|4 | 4\|4\|4\|4 | 8\|8\|8\|8 |
| CFR3pH | Cefuroxime | pH | sox | 4\|4\|4\|4 | 4\|4\|4\|4 | 4\|4\|4\|4 | 8\|8\|8\|8 |
| CFR6pH | Cefuroxime | pH | mar | 4\|4\|4\|4 | 4\|4\|4\|4 | 4\|4\|4\|4 | 8\|8\|8\|8 |
| CFR5Temp | Cefuroxime | Temp | acr | 4\|4\|4\|4 | 4\|4\|4\|4 | 4\|4\|4\|4 | 8\|8\|8\|8 |
| CHL1Bile | Chloramphenicol | Bile | mar | 4\|4\|4\|4 | 4\|4\|4\|4 | 4\|4\|4\|4 | 8\|8\|8\|8 |
| CHL2Bile | Chloramphenicol | Bile | mar | 4\|4\|4\|4 | 4\|4\|4\|4 | 4\|4\|4\|4 | 8\|8\|8\|8 |
| CHL3Bile | Chloramphenicol | Bile | mar | 4\|4\|4\|4 | 4\|4\|4\|4 | 4\|4\|4\|4 | 8\|8\|8\|8 |
| CHL1pH | Chloramphenicol | pH | mar | 4\|4\|4\|4 | 4\|4\|4\|4 | 4\|4\|4\|4 | 8\|8\|8\|8 |
| CHL5pH | Chloramphenicol | pH | acr | 4\|4\|4\|4 | 4\|4\|4\|4 | 4\|4\|4\|4 | 8\|8\|8\|8 |
| GEN1Basal | Gentamicin | Basal | rib | 4\|4\|4\|4 | 4\|4\|4\|4 | 4\|4\|4\|4 | 8\|8\|8\|8 |
| GEN3Basal | Gentamicin | Basal | cpx | 4\|4\|4\|4 | 4\|4\|4\|4 | 4\|4\|4\|4 | 8\|8\|8\|8 |
| GEN1Bile | Gentamicin | Bile | rib | 4\|4\|4\|4 | 4\|4\|4\|4 | 4\|4\|4\|4 | 8\|8\|8\|8 |
| GEN4Bile | Gentamicin | Bile | ubi | 4\|4\|4\|4 | 4\|4\|4\|4 | 4\|4\|4\|4 | 8\|8\|8\|8 |
| GEN5Bile | Gentamicin | Bile | hem | 4\|4\|4\|4 | 4\|4\|4\|4 | 4\|4\|4\|4 | 8\|8\|8\|8 |
| GEN1pH | Gentamicin | pH | cpx | 4\|4\|4\|4 | 4\|4\|4\|4 | 4\|4\|4\|4 | 8\|8\|8\|8 |
| GEN4pH | Gentamicin | pH | nuo | None | None | None | 2\|2\|3\|2 |
| GEN6pH | Gentamicin | pH | hem | 3\|3\|3\|3 | 3\|2\|3\|2 | 3\|2\|3\|2 | 6\|5\|6\|5 |
| GEN1Temp | Gentamicin | Temp | rib | 4\|4\|4\|4 | 4\|4\|4\|4 | 4\|4\|4\|4 | 8\|8\|8\|8 |
| GEN4Temp | Gentamicin | Temp | ubi | 3\|3\|2\|3 | 2\|3\|2\|3 | 2\|3\|2\|3 | 5\|6\|4\|6 |
| GEN6Temp | Gentamicin | Temp | rib | 3\|3\|2\|2 | 2\|3\|2\|3 | 2\|3\|2\|3 | 5\|6\|4\|5 |
| STR1Basal | Streptomycin | Basal | rib | 4\|4\|4\|4 | 4\|2\|4\|4 | 4\|2\|4\|4 | 8\|7\|8\|8 |
| STR3Basal | Streptomycin | Basal | ubi | 4\|4\|4\|4 | 4\|3\|3\|4 | 4\|3\|3\|4 | 8\|7\|8\|8 |
| STR6Basal | Streptomycin | Basal | rib | 4\|4\|4\|4 | 4\|4\|4\|4 | 4\|4\|4\|4 | 8\|8\|8\|8 |
| STR1Bile | Streptomycin | Bile | atp | 4\|4\|4\|4 | 4\|4\|4\|4 | 4\|4\|4\|4 | 8\|8\|8\|8 |
| STR5Bile | Streptomycin | Bile | ubi | 4\|4\|4\|4 | 4\|4\|4\|4 | 4\|4\|4\|4 | 8\|8\|8\|8 |
| STR1pH | Streptomycin | pH | rps | 4\|4\|4\|4 | 4\|4\|3\|4 | 4\|4\|3\|4 | 8\|8\|8\|8 |
| STR2pH | Streptomycin | pH | rsm | 4\|4\|4\|4 | 4\|4\|4\|4 | 4\|4\|4\|4 | 8\|8\|8\|8 |
| STR4pH | Streptomycin | pH | rps | 4\|4\|4\|4 | 4\|4\|2\|4 | 4\|4\|2\|4 | 8\|8\|8\|8 |
| STR5pH | Streptomycin | pH | tuf | 2\|2\|2\|2 | 2\|2\|3\|2 | 2\|2\|3\|2 | 4\|4\|5\|4 |
| STR1Temp | Streptomycin | Temp | atp | 3\|3\|3\|3 | 3\|3\|3\|3 | 3\|3\|3\|3 | 6\|6\|7\|7 |
| TRM2Basal | Trimethoprim | Basal | fol | 4\|4\|4\|4 | None | None | 8\|8\|8\|8 |
| TRM3Basal | Trimethoprim | Basal | pho | 4\|4\|4\|4 | 4\|4\|4\|4 | 4\|4\|4\|4 | 8\|8\|8\|8 |
| TRM5Basal | Trimethoprim | Basal | fol | 4\|4\|4\|4 | None | None | 8\|8\|8\|8 |
| TRM1Bile | Trimethoprim | Bile | fol | 4\|4\|4\|4 | 4\|4\|3\|4 | 4\|4\|3\|4 | 8\|8\|8\|8 |
| TRM3Bile | Trimethoprim | Bile | mgr | 4\|4\|4\|4 | 4\|4\|4\|4 | 4\|4\|4\|4 | 8\|8\|8\|8 |
| TRM4Bile | Trimethoprim | Bile | pho | 3\|2\|2\|2 | None | None | 5\|5\|3\|4 |
| TRM6Bile | Trimethoprim | Bile | pho | 4\|4\|4\|4 | 4\|4\|4\|4 | 4\|4\|4\|4 | 8\|8\|8\|8 |
| TRM3pH | Trimethoprim | pH | fol | 4\|4\|4\|4 | 4\|4\|4\|4 | 4\|4\|4\|4 | 8\|8\|8\|8 |
| TRM5pH | Trimethoprim | pH | pho | 4\|4\|4\|4 | 4\|4\|4\|4 | 4\|4\|4\|4 | 8\|8\|8\|8 |
| TRM6pH | Trimethoprim | pH | fol | 4\|4\|4\|4 | 3\|4\|3\|4 | 3\|4\|3\|4 | 8\|8\|8\|8 |
| TRM1Temp | Trimethoprim | Temp | pho | 4\|4\|4\|4 | 4\|4\|4\|4 | 4\|4\|4\|4 | 8\|8\|8\|8 |
| TRM2Temp | Trimethoprim | Temp | pho | 4\|4\|4\|4 | 4\|4\|4\|4 | 4\|4\|4\|4 | 8\|8\|8\|8 |
| TRM3Temp | Trimethoprim | Temp | pho | 4\|4\|4\|4 | 4\|4\|4\|4 | 4\|4\|4\|4 | 8\|8\|8\|8 |
| TRM5Temp | Trimethoprim | Temp | pho | 4\|4\|4\|4 | 4\|4\|4\|4 | 4\|4\|4\|4 | 8\|8\|8\|8 |
| TRM6Temp | Trimethoprim | Temp | pho | 4\|4\|4\|4 | 4\|4\|4\|4 | 4\|4\|4\|4 | 8\|8\|8\|8 |
| Number of mutants included in models | | | | 56 | 53 | 53 | 57 |
| Number of data points used in models | | | | 858 | 805 | 805 | 1723 |
