# Supplementary material for: Collateral Sensitivity Interactions between Antibiotics Depend on Local Abiotic Conditions
Source: mSystems. 2021 Nov 30;6(6):e01055-21. doi: 10.1128/mSystems.01055-21 (PMC8631318; doi:10.1128/mSystems.01055-21)
Supplement: TABLE S3 [file msystems.01055-21-st003.docx]

| Selection Drug | Assay Drug | Phenotype | **Model A : Genotype** | | | | | **Model B: Selection Conditions** | | | | | |
| --- | --- | --- | --- | --- | --- | --- | --- | --- | --- | --- | --- | --- | --- |
|  |  |  | Random Effects | | Fixed Effects | | | Random Effects | | Fixed Effects | | |  |
|  |  |  | Strain | Strain : Block | Assay Environment | Genotype | Interaction | Strain | Strain : Block | Assay Environment | Selection Environment | Sympatry |  |
| Cefuroxime | Cefuroxime | IC_90_ |  |  |  |  |  |  |  |  |  |  |  |
| Gentamicin | Gentamicin | IC_90_ |  |  |  |  |  |  |  |  |  |  |  |
| Streptomycin | Streptomycin | IC_90_ |  |  |  |  |  |  |  |  |  |  |  |
| Trimethoprim | Trimethoprim | IC_90_ |  |  |  |  |  |  |  |  |  |  |  |
| Cefuroxime | Cefuroxime | GASC |  |  |  |  |  |  |  |  |  |  |  |
| Gentamicin | Gentamicin | GASC |  |  |  |  |  |  |  |  |  |  |  |
| Streptomycin | Streptomycin | GASC |  |  |  |  |  |  |  |  |  |  |  |
| Trimethoprim | Trimethoprim | GASC |  |  |  |  |  |  |  |  |  |  |  |
| Cefuroxime | Gentamicin | IC_90_ |  |  |  |  |  |  |  |  |  |  |  |
| Gentamicin | Cefuroxime | IC_90_ |  |  |  |  |  |  |  |  |  |  |  |
| Streptomycin | Tetracycline | IC_90_ |  |  |  |  |  |  |  |  |  |  |  |
| Trimethoprim | Nitrofurantoin | IC_90_ |  |  |  |  |  |  |  |  |  |  |  |
| Cefuroxime | None | Cost |  |  |  |  |  |  |  |  |  |  |  |
| Gentamicin | None | Cost |  |  |  |  |  |  |  |  |  |  |  |
| Streptomycin | None | Cost |  |  |  |  |  |  |  |  |  |  |  |
| Trimethoprim | None | Cost |  |  |  |  |  |  |  |  |  |  |  |
